# Cryo-EM structure of adeno-associated virus 4 at 2.2 Å resolution

**DOI:** 10.1107/S2059798322012190

**Published:** 2023-01-20

**Authors:** Grant Zane, Mark Silveria, Nancy Meyer, Tommi White, Rui Duan, Xiaoqin Zou, Michael Chapman

**Affiliations:** a Department of Biochemistry, University of Missouri, Columbia, MO 65211, USA; b Center for Spatial Systems Biomedicine, Oregon Health Sciences University, Portland, Oregon, USA; c Bayer Crop Science, Bayer (United States), Chesterfield, MO 63017, USA; d Electron Microscopy Core, University of Missouri, Columbia, MO 65211, USA; e Dalton Cardiovascular Center, University of Missouri, Columbia, MO 65211, USA; f Department of Physics, University of Missouri, Columbia, MO 65211, USA; g Institute for Data Science and Informatics, University of Missouri, Columbia, MO 65211, USA; Thomas Jefferson University, USA

**Keywords:** AAV4, cryo-electron microscopy, adeno-associated virus receptor, gene therapy

## Abstract

Adeno-associated virus (AAV) is the vector of choice for several approved gene-therapy treatments and is the basis for many ongoing clinical trials. Various strains of AAV exist (referred to as serotypes), each with their own transfection characteristics. Here, a high-resolution cryo-electron microscopy structure (2.2 Å) of AAV serotype 4 (AAV4) is presented. The receptor responsible for transduction of the AAV4 clade of AAV viruses (including AAV11, AAV12 and AAVrh32.33) is unknown. Other AAVs interact with the same cell receptor, adeno-associated virus receptor (AAVR), in one of two different ways. AAV5-like viruses interact exclusively with the polycystic kidney disease-like 1 (PKD1) domain of AAVR, while most other AAVs interact primarily with the PKD2 domain. A comparison of the present AAV4 structure with prior corresponding structures of AAV5, AAV2 and AAV1 in complex with AAVR provides a foundation for understanding why the AAV4-like clade is unable to interact with either PKD1 or PKD2 of AAVR. The conformation of the AAV4 capsid in variable regions I, III, IV and V on the viral surface appears to be sufficiently different from AAV2 to ablate binding with PKD2. Differences between AAV4 and AAV5 in variable region VII appear to be sufficient to exclude binding with PKD1.

## Abstract 2

An updated structure of adeno-associated virus serotype 4 (AAV4) is presented with a comparison to receptor-bound structures (AAV2 and AAV5) to predict why AAV4 does not bind to the adeno-associated virus receptor.

## 1. Introduction

Adeno-associated virus (AAV) is an icosahedral, non-enveloped, single-stranded DNA virus which can infect humans. AAV was initially touted as a potential vector for gene therapy, because it is genetically amenable and the wild-type virus had not been associated with any diseases (Naso *et al.*, 2017 ▸). It is currently the gene-therapy delivery vector of choice in treatments for a number of monogenic genetic diseases such as spinal muscular atrophy (SMA; Mendell *et al.*, 2021 ▸; Hoy, 2019 ▸). When used as a vector to deliver gene therapy, the genome of the virus is replaced with the gene of interest, resulting in a recombinant AAV (rAAV). After some debate, the consensus is that the risk of inducing hepato­cellular carcinoma is minimal in those without liver disease (de Jong & Herzog, 2021 ▸; Dalwadi *et al.*, 2021 ▸), and no other disease has been connected with AAV infection. However, concern remains that particularly high vector loads of rAAV have been linked to fatal immunotoxicity in some test subjects (Morales *et al.*, 2020 ▸; Philippidis, 2020 ▸). Therefore, research to explore how various AAV strains (also referred to as serotypes) interact with cell receptors will hopefully add to our understanding of serotype-specific phenotypes and lay a foundation for improvement of the efficiency and specificity of vectors in different targeting modalities.

The AAV genome codes for three capsid proteins (VP1, VP2 and VP3). The DNA coding for the capsid proteins contains three different start codons such that all transcripts share the same C-terminal sequence of the VP3 protein (consisting of ∼540 amino acids; Muzyczka & Berns, 2001 ▸). VP1 contains an ∼195-amino-acid extension beyond the N-terminus of VP3, while VP2 starts ∼60 amino acids upstream of the N-terminus of VP3. The ∼60-amino-acid sequence found in both VP1 and VP2 is referred to as the VP1/2 common sequence. The estimated ratio of the three VP proteins in a mature capsid is 1:1:10. The portion of VP1 that is unique to VP1 (∼135 amino acids) is referred to as the VP1 unique region (VP1u). VP1u contains a phospholipase A_2_ (PLA_2_) domain (Bleker *et al.*, 2005 ▸; Girod *et al.*, 2002 ▸; Popa-Wagner *et al.*, 2012 ▸), along with a putative nuclear-localization signal (NLS; Kurian *et al.*, 2019 ▸; Johnson *et al.*, 2010 ▸). In a mature capsid, VP1u and the VP1/2 common region are situated inside the capsid (with the genomic ssDNA) in locations that have not been apparent by X-ray crystallography and have been hinted at in cryo-electron microscopy (cryo-EM) of some AAVs but not others (Kronenberg *et al.*, 2001 ▸, 2005 ▸; Gerlach *et al.*, 2011 ▸; Hu, Silveria, Chapman *et al.*, 2022 ▸). It is not unexpected that the VP1u and VP1/2 regions have been refractory to high-resolution structure determination, because they are present in only 10% and 20%, respectively, of the capsid subunits that must be 60-fold symmetry-averaged in most approaches to structure determination. In addition to the resulting signal dilution due to symmetry averaging, they may also be in a disordered state. During endosomal trafficking of the virus from the cell surface to the nucleus, VP1u and the VP1/2 common region become exposed and have been shown to be important for efficient transduction (Sonntag *et al.*, 2006 ▸; Johnson *et al.*, 2010 ▸; Lins-Austin *et al.*, 2020 ▸; Kronenberg *et al.*, 2005 ▸; Venkatakrishnan *et al.*, 2013 ▸; Madigan *et al.*, 2020 ▸).

Briefly, AAV infection occurs through the following steps. Firstly, the virus binds to serotype-specific glycan attachment factors such as heparin sulfate or sialic acid on the surface of the cell (Summerford & Samulski, 1998 ▸; Walters *et al.*, 2001 ▸). Next, the virus interacts with a protein surface receptor (AAVR for all serotypes except AAV4 clade members) to facilitate entry of the virus into an endosome (Pillay *et al.*, 2016 ▸). In the endosome the pH decreases, which results in destabilization of the virus and VP1u is exuded (Lins-Austin *et al.*, 2020 ▸). Concomitant with the drop in pH, the endosomal calcium concentration also rises with the assistance of SPCA1, an ATP-powered calcium pump encoded by the ATP2C1 gene of the host (Madigan *et al.*, 2020 ▸). *In vitro*, VP1u can be exposed by heat treatment (Sonntag *et al.*, 2006 ▸), but the combination of lower pH, lower calcium concentration and other factors that corresponds to the physiological trigger is not yet clear (Lins-Austin *et al.*, 2020 ▸). The presence of host protein GPR108 enhances transduction by AAV (except for AAV5) downstream of AAVR binding and presumably during trafficking or uncoating (Dudek *et al.*, 2020 ▸). Chimeric mutants show that GPR108-independence is conferred by the AAV5 VP1u, suggesting that as VP1u is sequestered inside the capsid until late in endosomal trafficking, the GPR108 interaction may be important in endosomal escape. The virus is then trafficked to the nucleus with the assistance of the NLS regions located on the VP1/2 common region (Johnson *et al.*, 2010 ▸), where the viral genome (or, for rAAV, the transgene) is transported into the nucleus and expressed.

**Figure 1:**
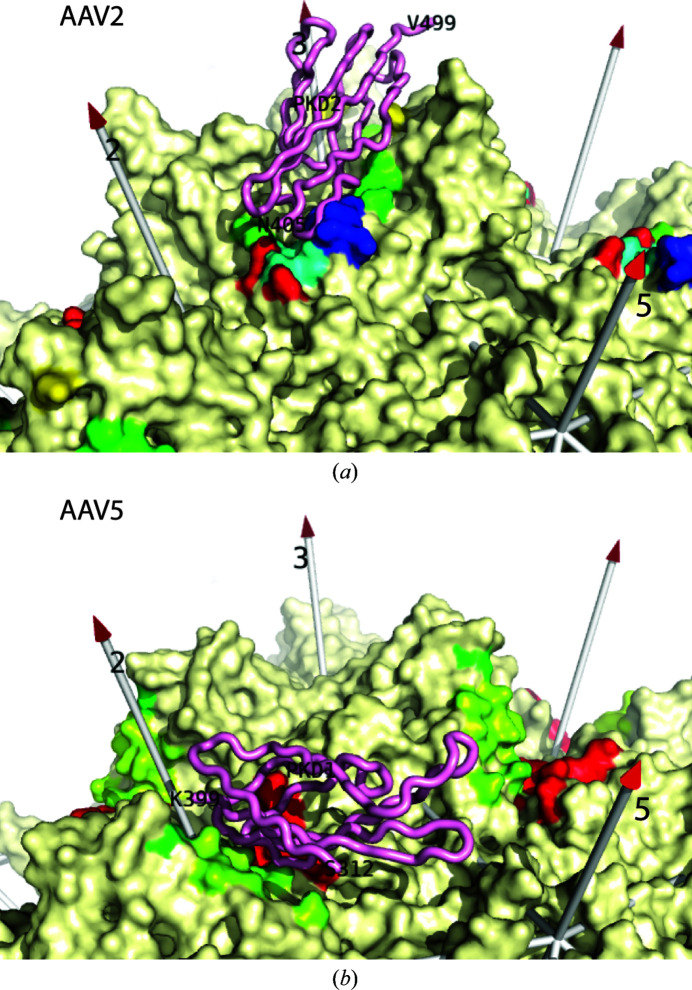
Binding sites for AAVR on AAV2 and AAV5. Single copies of the PKD2 and PKD1 domains of AAVR (pink backbone) are shown as they are bound in structures of the AAV2 (top) and AAV5 (bottom) complexes, respectively (Meyer *et al.*, 2019 ▸; Silveria *et al.*, 2020 ▸). The viral surfaces (cream) are viewed with a similar perspective as in Fig. 5 ▸. When within 4.5 Å of receptor atoms, AAV amino acids are rainbow-colored by residue number from blue to red so that their location within a subunit can be registered by the corresponding color in Fig. 5 ▸.

Differential patterns of molecular interactions between the various serotypes and AAVR are emerging. AAVR is a C-terminally membrane-anchored receptor with an ecto­domain consisting of an N-terminal signal peptide, a MANEC (motif at the N-terminus containing eight cysteines) domain and five non-identical polycystic kidney disease (PKD) domains (Poon *et al.*, 2011 ▸). The tandem PKD domains are named PKD1 through PKD5 (from N-terminal to C-terminal). Initial studies of AAVR (Pillay *et al.*, 2017 ▸) showed that AAV2 was dependent primarily upon PKD2 for cell transduction and secondarily on PKD1 (in an accessory role), but was not dependent on other PKD domains. AAV5 required PKD1, but not any other PKD domains, including PKD2. The two PKD domains that interact with AAV (PKD1 and PKD2) share modest sequence identity (30%) and the same immuno­globulin-like fold (two layers of β-sheet). Cryo-EM structures of AAV2 in complex with either all five PKD domains (PKD15; Meyer *et al.*, 2019 ▸) or with only the first two domains (PKD12) support the observation that AAV2 interacts primarily with PKD2. In both studies AAV2 interacts tightly and specifically with the PKD2 domain. Other domains of AAVR lack direct interactions with AAV2 and are too flexibly oriented to be resolved by high-resolution cryo-EM, but PKD1 has recently been located in several configurations by cryo-electron tomography (cryo-ET; Hu, Silveria, Zane *et al.*, 2022 ▸). In complex with AAV1, PKD2 is bound as with AAV2 (Zhang, Xu *et al.*, 2019 ▸). By contrast, the high-resolution cryo-EM structure of AAV5 in complex with PKD15 (Zhang, Xu *et al.*, 2019 ▸) or PKD12 (Silveria *et al.*, 2020 ▸) shows strongly bound PKD1. PKD2 is now only seen at lower resolution by cryo-ET (Hu, Silveria, Zane *et al.*, 2022 ▸) in several orientations extending away from the virus. Note that the tight PKD1 binding site on AAV5 is distinct from the PKD2 site on AAV2 (Fig. 1 ▸). Overlay of the AAV2 and AAV5 complexes shows that it would not be possible to connect the observed PKD1 and PKD2 domains into a single polypeptide in a hypothetical unobserved AAV2 complex with the PKD1 domain oriented as in the AAV5 complex (Silveria *et al.*, 2020 ▸).

In the current work, we focus on AAV4 since it is a serotype that is not dependent on AAVR for successful cellular transduction. Cryo-EM is used to extend the resolution of the AAV4 structure beyond that of the prior crystallographic structure at 3.2 Å resolution (PDB entry 2g8g; Govindasamy *et al.*, 2006 ▸). The crystal structure was a seminal advance in 2006 because, with just 59% sequence identity to the then only high-resolution structure, the type-species AAV2 (Xie *et al.*, 2002 ▸), it highlighted the diversity possible among structures of human AAVs. It provided an opportunity to define surface segments of the primary sequence as variable regions in structure (VR-I through VR-IX) that would, in time, become associated with distinctive functional phenotypes of AAV serotypes. The AAV4 crystal structure had been preceded by a cryo-EM structure (Padron *et al.*, 2005 ▸) at 13 Å resolution, which was impressive in 2005, but was then sufficient only to dock in an AAV2-based pseudo-atomic model. A ‘cryo-EM resolution revolution’, based on improved microscope stability, fast and sensitive direct detectors and improved computational methods (Cheng, 2015 ▸; Scheres, 2016 ▸; Baldwin *et al.*, 2018 ▸), has made possible the determination of AAV structures at higher resolution than by crystallography (Tan *et al.*, 2018 ▸; Xie *et al.*, 2020 ▸). Fourier image theory tells us that the information content of a 3D structure at 2.2 Å resolution is threefold higher than at 3.2 Å resolution. Thus, with the goal of understanding how inter-serotype structural differences might underpin an ability to use AAVR for cellular entry, there was a need for an AAV4 structure with a precision comparable to those of AAVR-binding serotypes (Silveria *et al.*, 2020 ▸; Meyer *et al.*, 2019 ▸). With structures available of several AAV4 homologs (AAVrh32.33, AAV11 and AAV12; Mietzsch *et al.*, 2021 ▸; Mikals *et al.*, 2014 ▸) we can explore the features, conserved among AAVR-independent serotypes, that are significantly different compared with AAVR-binding serotypes.

With the assumption that the interactions between AAV and AAVR are conserved within closely related serotypes, we sought to determine which features of AAV are important for binding to AAVR. For the PKD2 interface, structures of receptor complexes were available for AAV2 (and AAV1) (Meyer *et al.*, 2019 ▸; Zhang, Cao *et al.*, 2019 ▸; Zhang, Xu *et al.*, 2019 ▸) and we could also superimpose structures of uncomplexed AAV capsids known or presumed to interact analogously (Pillay *et al.*, 2017 ▸; Dudek *et al.*, 2018 ▸). For the PKD1 interface, receptor complexes were available for AAV5 (Zhang, Xu *et al.*, 2019 ▸; Silveria *et al.*, 2020 ▸) and homologous serotype structures could similarly be superimposed. An earlier comparison of the 2.4 Å resolution AAV2–AAVR complex with the 3.2 Å resolution uncomplexed AAV4 structure provided a preliminary rationale for the incompatibility of AAV4 with a mode of AAVR binding like that of AAV2 (Meyer *et al.*, 2019 ▸). Now, with a high-resolution AAV4 structure in hand, the incompatibilities of AAV4 with PKD2 binding are detailed and, for the first time, incompatibilities with PKD1 binding (as in AAV5) are elucidated. AAV2 and AAV5 represent the two distinct modes of AAVR binding, so comparisons with both are needed to understand why the AAV4 clade differs from all other primate AAVs in lacking interactions with AAVR (Pillay *et al.*, 2017 ▸; Dudek *et al.*, 2018 ▸).

## 2. Materials and methods

### 2.1. Expression and purification of AAV4

Virus-like particles (VLPs) for AAV4 were expressed in *Spodoptera frugiperda* Sf9 cells from a polyhedron H promoter (P_polH_) on a bacmid. The bacmid was constructed in *Escherichia coli* DH10Bac cells following transformation with the pFastBac LIC cloning vector (4A; Addgene plasmid #30111) containing a cloned copy of the AAV4 VP1 gene. The VP1 gene was cloned into the destination plasmid using ligation-independent cloning (see Supplementary Table S1; Li & Elledge, 2007 ▸). The start codon for VP1 was mutated to ACG in order to downregulate expression of VP1 to accommodate the desired ratio of the three capsid proteins (1:1:10), as performed elsewhere (Silveria *et al.*, 2020 ▸; Urabe *et al.*, 2002 ▸). Sequencing of the cloned gene was performed at the University of Missouri DNA core facility and compared with the published sequence of AAV4 (accession No. NC_001829). Following expression in Sf9 cells, the VLPs were isolated and purified as described previously (Meyer *et al.*, 2020 ▸) except that purification by heparin affinity, which is expected to work only for serotypes with heparan sulfate attachment factors, was replaced by additional iterations of caesium chloride gradient ultracentrifugation (Silveria *et al.*, 2020 ▸). AAV4 VLP was then dialyzed into HN buffer (25 m*M* HEPES, 150 m*M* sodium chloride pH 7.4) and used in additional experiments.

### 2.2. Single-particle cryo-EM sample preparation and imaging

Samples of dialyzed AAV4 VLP (2 µl at 0.33 mg ml^−1^) were placed on glow-discharged EM grids (copper grids with lacey carbon; Ted Pella catalog No. 01824) and allowed to adhere for 2 min. Using an FEI Vitrobot Mark IV, the sample was blotted (blot force 4; time 2 s; temperature 25°C; humidity 100%) and plunged into liquid ethane. Cryo-EM images were recorded at the Pacific Northwest Cryo-EM Center on a 300 keV Titan Krios electron microscope (Thermo Scientific) equipped with a Gatan K3 detector, and BioQuantum energy filter 4809 image stacks were collected at a super-resolution pixel size of 0.256 Å (a nominal magnification of 165 000×). Each stack comprised 80 frames and a total dose of 47 e^−^ Å^−2^ per movie, with defocus varying between −3.9 and 1.0 µm (Table 1 ▸).

### 2.3. Image processing

The cryo-EM images were processed using *RELION* 3.1.1 (Scheres, 2012 ▸). The movie files were motion-corrected using the *MotionCor*2-like algorithm in *RELION*, and CTF estimation was performed within *RELION* using *CTFFIND*4.1 (Rohou & Grigorieff, 2015 ▸). Picking templates were generated in *RELION* by 2D classification of LoG-picked particles followed by autopicking of the entire data set. All extracted particles were then sorted into 20 2D classes, and the 18 best classes were selected for 3D refinement with the application of *I*1 symmetry. Post-processing in *RELION* included both per-particle CTF estimation and per-particle motion correction. Subsequent final refinement and masking resulted in a Coulombic potential map of 2.2 Å resolution by Fourier shell correlation (FSC) gold-standard estimation (Supplementary Fig. S1).

### 2.4. Atomic modeling and refinement

*RSRef* (Chapman *et al.*, 2013 ▸) was used to calibrate the magnification of the AAV4 Coulombic potential map against the 3.2 Å resolution structure of AAV4 obtained by X-ray crystallography (PDB entry 2g8g; Govindasamy *et al.*, 2006 ▸). The magnification was adjusted by 0.41%. The published AAV4 crystal structure was overlaid on the adjusted AAV4 electron-density map; model backbone and rotamer adjustments were performed manually within *Coot* 0.8.9.2 (Emsley *et al.*, 2010 ▸). Such interactive model building was iterated with optimization using *RSRef*-embedded *CNS* (Chapman *et al.*, 2013 ▸; Brünger *et al.*, 1998 ▸) to perform stereochemically restrained, all-atom real-space refinement (parameterized in Cartesian space). In the penultimate iteration, H atoms were added temporarily for more aggressive improvement of van der Waals interactions. The final two iterations included the refinement of individual atomic *B* factors, subject to a restraint that the r.m.s. difference between bonded neighbors was ∼1 Å^2^. The final correlation of the model to the experimental 3D Coulombic potential map was 0.86 for all map grid points within 2.4 Å of any non-H atom.

### 2.5. Molecular-dynamics simulations

To investigate why AAV4 does not bind AAVR and why the interface might not be sufficiently flexible to accommodate the unique characteristics of AAV4, molecular-dynamics (MD) simulations were performed on models of the AAV4 complex with PKD1 or PKD2. AAV4 was represented by the subunit dimer proximal to AAVR. PKD1 was docked to AAV4 by superimposition of the PKD1–AAV5 cryo-EM structure (PDB entry 7kpn; Silveria *et al.*, 2020 ▸) using *PyMOL* (version 2.5.2; Schrödinger). This was rigid-body superimposition except that the side chain of AAV4 Arg723 was changed to the rotamer found in the AAV5–PKD1 complex. PKD2 was similarly docked to AAV4, this time by superimposing the PKD2–AAV2 cryo-EM structure (PDB entry 6nz0; Meyer *et al.*, 2019 ▸). For comparison of the free energies of binding and structural fluctuations, MD simulations on these AAV4 models were compared with simulations of a PKD1–AAV5 dimer extracted from the cryo-EM structure of the complex (PDB entry 7kpn). AAV9 was also used as a control, starting with the unbound crystal structure (PDB entry 3ux1; DiMattia *et al.*, 2012 ▸), docking PKD2 as above and then comparing the results of MD simulation with the recent experimental structure of PKD2–AAV9 (PDB entry 7wjx; Xu *et al.*, 2022 ▸).

To perform MD simulations, each of the four modeled complexes was minimized with *UCSF Chimera* (Pettersen *et al.*, 2004 ▸) to remove atomic clashes, processed using the Protein Preparation Workflow in *Maestro* (version 13.3.121; Schrödinger) to determine amino-acid protonation states and then processed with the *Leap* module to link the disulfide bonds. The complexes were then solvated in a periodic box of TIP3P water (Jorgensen *et al.*, 1983 ▸) with the minimum distance between the complex and the water box surface at 12 Å. Na^+^ ions were also added to neutralize the system. The simulation parameters for the complexes were provided in AMBER FF14SB (Maier *et al.*, 2015 ▸). The systems were minimized in two steps. They were optimized for 1000 steps by the steepest-descent method and 3000 steps by the conjugate-gradient method. The complexes were restrained with a weight of 500 kcal mol^−1^ Å^−2^. The complexes and solvent were then minimized for 10 000 steps via steepest descent, followed by conjugate gradient until the energy gradient of the system converged to 0.01 kcal mol^−1^ Å^−1^. Afterwards, the systems were heated from 0 to 300 K in 100 ps, and 30 ns of simulation was then run with a time step of 2 fs. Langevin dynamics (Pastor *et al.*, 1988 ▸) were applied to regulate the temperature of the system during MD simulation, with a collision frequency of 4.0 ps^−1^. The particle mesh Ewald method (Essmann *et al.*, 1995 ▸) was used to treat long-range electrostatic interactions and a 12 Å cutoff was used for any nonbonded van der Waals (vdW) interactions. The *SHAKE* algorithm (Ryckaert *et al.*, 1977 ▸) was used to restrain bonds involving H atoms. All of the simulations were performed using the *Amber*16 package (Case *et al.*, 2016 ▸) on a Dell PowerEdge R740XD server (three Nvidia Tesla V100 32G Passive GPUs).

The free energy of binding was estimated for each complex using the MM/PBSA method (Kollman *et al.*, 2000 ▸; Massova & Kollman, 2000 ▸) with 1500 frames from the last 15 ns simulation extracted at an interval of 10 ps. The entropic contributions were estimated using the interaction entropy (Duan *et al.*, 2016 ▸). All of the values of the interaction entropies were weighted by a factor of 0.4 to make them comparable to the enthalpic energies.

### 2.6. Thermal shift assays

Thermal shift assays were performed with VLPs of serotypes AAV2, AAV4 and AAV5, and these were compared with one another. Each 20 µl sample contained 0.125 mg VLP per millitre (HEPES-buffered), 1× sample dye and 1× supplied buffer (Protein Thermal Shift Dye Kit; Thermo Fisher 446-1146). Each sample was run in triplicate. The samples were prepared on ice and incubated in the dark for 20 min before being subjected to thermal denaturation on a QS3 thermocycler (Applied Biosystems). The samples were incubated at the designated temperature (20–99.9°C) for 1 min, read for fluorescence, heated by 0.3°C and repeated. The data were processed using a local interface to a custom thermal shift assay script [https://beamerlab.shinyapps.io/software/; ‘TSA (1-X, many-Y)’] based upon the calculations described elsewhere (Andreotti *et al.*, 2015 ▸).

### 2.7. ELISA binding assay

ELISA experiments were performed as described previously (Silveria *et al.*, 2020 ▸). Two ELISA experiments were conducted: one to test for the binding of PKD12 to AAV4 and another to show that the AAV4-specific antibody (ADK4), but not the AAV5-specific antibody (ADK5b), could bind to AAV4 VLP. To check for the binding of PKD12 to AAV4, various AAV serotypes (AAV2, AAV4 and AAV5) were adhered to a 96-well ELISA plate (Costar, 9018), bovine serum albumin (BSA; Thermo Fisher, BP9703-100) was used to block the plate, a sample of 6×His-tagged PKD12 was allowed to bind to the virus and an HRP-conjugated antibody (Proteintech, 66005) was allowed to bind to the His-tagged ligand.

AAV4 and AAV5 VLP preparations were validated using the serotype-specific monoclonal antibodies ADK4 (Progen, 610147) and ADK5b (Origene, AM09121PU-N). VLPs were adhered to the ELISA plate, the wells were blocked with BSA, the primary mouse antibody was allowed to bind to AAV and the secondary antibody was then added (HRP-conjugated, goat-derived; Thermo Fisher, 626520). Following binding of the HRP-conjugated antibody, the HRP substrate (Abcam, 171523) was added followed by hydrochloric acid (Thermo Fisher, A144C-212) to quench the reaction. The signal was read at 450 nm (BioTek, Synergy H1). All samples were run in triplicate.

### 2.8. Structures analyzed

The structures of the homologous AAVs were obtained from the PDB. For AAV1 bound to PKD15, PDB entry 6jcq (Zhang, Xu *et al.*, 2019 ▸) was used. For AAV2 bound to PKD12, PDB entry 6nz0 (Meyer *et al.*, 2019 ▸) was used. For AAV5 bound to PKD12, PDB entry 7kpn (Silveria *et al.*, 2020 ▸) was used. For AAV4, PDB entry 7thr, determined in this study, was used. For AAV7, PDB entry 7l5q (Mietzsch *et al.*, 2021 ▸) was used. For AAV8, PDB entry 2qa0 (Nam *et al.*, 2007 ▸) was used. For AAV9, PDB entry 3ux1 (DiMattia *et al.*, 2012 ▸) was used. For AAV11, PDB entry 7l6f (Mietzsch *et al.*, 2021 ▸) was used. For AAV12, PDB entry 7l6b (Mietzsch *et al.*, 2021 ▸) was used. For AAVrh32.33, PDB entry 4iov (Mikals *et al.*, 2014 ▸) was used. The phylogenetic tree was constructed with *ClustalX* (Larkin *et al.*, 2007 ▸).

## 3. Results and discussion

### 3.1. Biophysical and biochemical confirmation of AAV4

AAV4 VLPs were validated by comparison to the expected characteristics: thermal denaturation (thermal shift assay; Supplementary Table S2) and recognition by specific antibodies (ELISA). As expected, the AAV4 VLP reacted with monoclonal ADK4 and not with the AAV5-specific antibody ADK5b (data not shown).

### 3.2. AAV4 is not bound by AAVR

Transduction data for AAV4 show no dependence upon AAVR (Dudek *et al.*, 2018 ▸), which suggests that AAV4 might not bind to AAVR. This could also merely reflect the presence of a dominant alternative means of entry. Virus-overlay assays had previously shown no evidence of binding between AAV4 and fragments of AAVR (PKD1, PKD2, PKD3 or PKD15) that included all plausible binding domains (Dudek *et al.*, 2018 ▸). However, these negative results were treated with an abundance of caution because a viral overlay assay assumes that interactions remain possible in the environment of a denaturing gel, and this is not always true. Thus, AAVR–AAV4 binding was additionally measured by ELISA using the PKD12 construct (containing the first two PKD domains), for which the positive results with AAV2 and AAV5 provide a robust control (Supplementary Fig. S2). Again, AAV4 does not appear to interact with AAVR.

### 3.3. High-resolution structure of native AAV4

**Figure 2:**
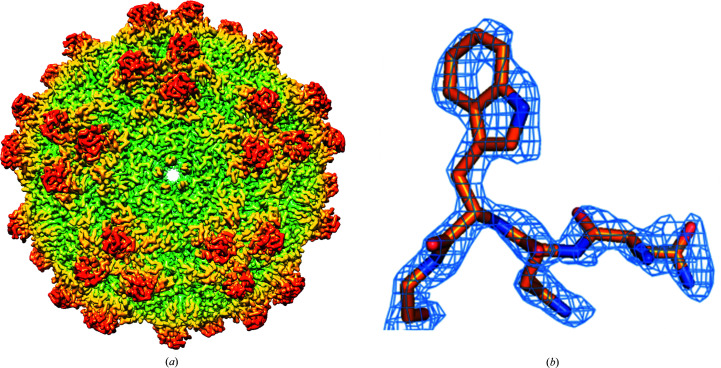
(*a*) Cryo-EM reconstruction of AAV4 at 2.21 Å resolution, color-coded by distance from the center. The view is down one of 12 pores running along fivefold axes of symmetry. Groups of three spikes related by threefold axes of symmetry encircle the fivefold. (*b*) Coulombic potential for AAV4 residues Asn293–Gly296.

The prepared AAV4 VLP was imaged by electron cryo-microscopy (cryo-EM) and the reconstruction was refined to 2.21 Å resolution, according to the gold-standard FSC curve cutoff (Supplementary Fig. S1). The quality of the 3D reconstruction is illustrated in Fig. 2 ▸, enabling the building of an atomic model with good stereochemistry and a map–model cross-correlation coefficient of 0.87. Full statistics are given in Table 1 ▸.

**Figure 3:**
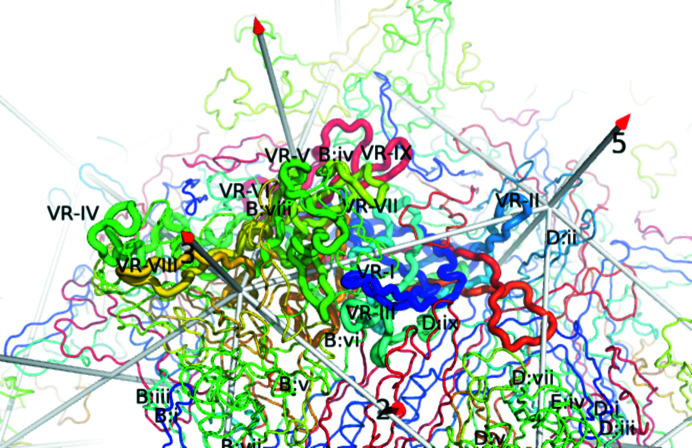
Structural context of the variable regions (VR) of the major capsid protein. A central VP3 (thick tubes) is surrounded by symmetry neighbors (thin tubes). The view is from outside the capsid, looking down an icosahedral twofold, with a threefold to the left and a fivefold to the right. Viewed from the exterior, the conserved β-barrel is hidden beneath the outer surface loops that dominate the foreground. Each subunit is rainbow-colored from the N-terminus (blue) to the C-terminus (red). The AAV4 cryo-EM structure is annotated by sequence-variable region (VR; Govindasamy *et al.*, 2006 ▸). VR-IV through VR-VIII are all contributed by a long loop between β-strands G and H. VRs of neighboring subunits that are most closely intertwined are annotated in abbreviated form (chain ID:loop No.).

Refinement against the 2.2 Å resolution cryo-EM reconstruction brings appreciable improvement over the crystal structure at 3.2 Å resolution (Govindasamy *et al.*, 2006 ▸). A structural superimposition of the two structures (Supplementary Fig. S3) shows, not unexpectedly, that they share the same backbone topology, differing mostly in local details. The all-atom r.m.s.d. is 0.80 Å, 0.72 Å with flipping of pseudo-symmetric side chains and 0.48 Å for backbone only. Overall, when compared with the new 2.2 Å resolution reconstruction, the correlation coefficient is improved from 0.77 to 0.87 when the crystallographic structure is replaced with the new EM refinement. A residue-by-residue comparison (by backbone or side chain) between PDB entry 2g8g and the 2.2 Å resolution structure is shown in Supplementary Fig. S4. The map for the 2.2 Å resolution structure was of poorer quality in variable region 2 (VR-II). Higher than average *B* factors in both structures indicate disorder and higher uncertainty, contributing to the locally high r.m.s.d. values. VR-II forms a β-hairpin loop that lines the fivefold pore at the outer surface of the virus (Fig. 3 ▸). In related parvoviruses this region has been implicated in encapsidation of the viral DNA and in extrusion of the previously sequestered VP1u phospholipase A_2_ domain (Farr *et al.*, 2005 ▸, 2006 ▸; Bleker *et al.*, 2005 ▸, 2006 ▸; Plevka *et al.*, 2011 ▸), functions that would require conformational flexibility. Other surface loops might be expected to be less constrained in structure than the core of the β-barrel but, of the other variable regions, it is only VR-IV and VR-VII where the r.m.s.d. exceeds the subunit average (Supplementary Table S3). As discussed in the following sections, VR-IV is key in blocking interactions of AAV4 with AAVR PKD2 and VR-VII is key in blocking interactions with PKD1, with the updated coordinates clarifying the extent of steric conflict.

Despite the overall similarity in other regions, the r.m.s.d. for all backbone atoms exceeds that for C^α^ atoms alone (0.48 versus 0.36 Å), an indication that carbonyl group orientations are not well defined in the tubular backbone density that is typical at 3.2 Å resolution. The differences are greater in loops of irregular structure than in secondary structures where *a priori* information can be used to define conformation (Supplementary Fig. S4). By contrast, at 2.2 Å resolution ambiguities are resolved now that many of the carbonyl O atoms become discernible. The 2.2 Å resolution map also supports many other local improvements, particularly where side chains become well defined for the first time (examples are shown in Supplementary Fig. S5 and Supplementary Table S4).

Lastly, the current structure includes two magnesium ions and 219 water molecules per protein subunit (examples are shown in Supplementary Fig. S6). This compares with 15 water molecules in the 3.2 Å resolution structure. One of the ions (MG2 2000) had been modeled as water 10 in the 3.2 Å resolution structure, but the high-resolution map and coordination are more consistent with a cation, presumed to be Mg^2+^ from the buffer.

### 3.4. Structural superimposition of AAV4 on AAVR-bound AAV

We compared the current AAV4 structure with published AAVR–AAV structures to identify components of the AAV4 structure, at the amino-acid level, which render AAV4 incompatible with the binding of AAVR. The AAV4 structure was aligned with several AAVR-bound complexes using the align function within *PyMOL* (DeLano, 2002 ▸; Schrödinger). Additionally, serotypes from phylogenetically distinct clades as well as a few closely related to AAV4 (Supplementary Fig. S7) were included to discern any patterns that might predict binding to AAVR.

#### 3.4.1. Comparison of AAV4 at the PKD2-binding site as seen in AAV2 and AAV1

For AAV2, residues from VR-I, VR-III, VR-V, VR-VI, VR-VIII and VR-IX (plus a few others) are in contact with PKD2 (Zhang, Cao *et al.*, 2019 ▸; Meyer *et al.*, 2019 ▸; Table 2 ▸, Fig. 1 ▸ *a*). AAV4 and other serotypes were aligned with the structure of the AAV2–PKD12 complex. Each of the contact residues was examined for changes in steric hindrance, electrostatic charge or more subtle differences that could impact the ability of AAV4 to bind to AAVR.

**Figure 4:**
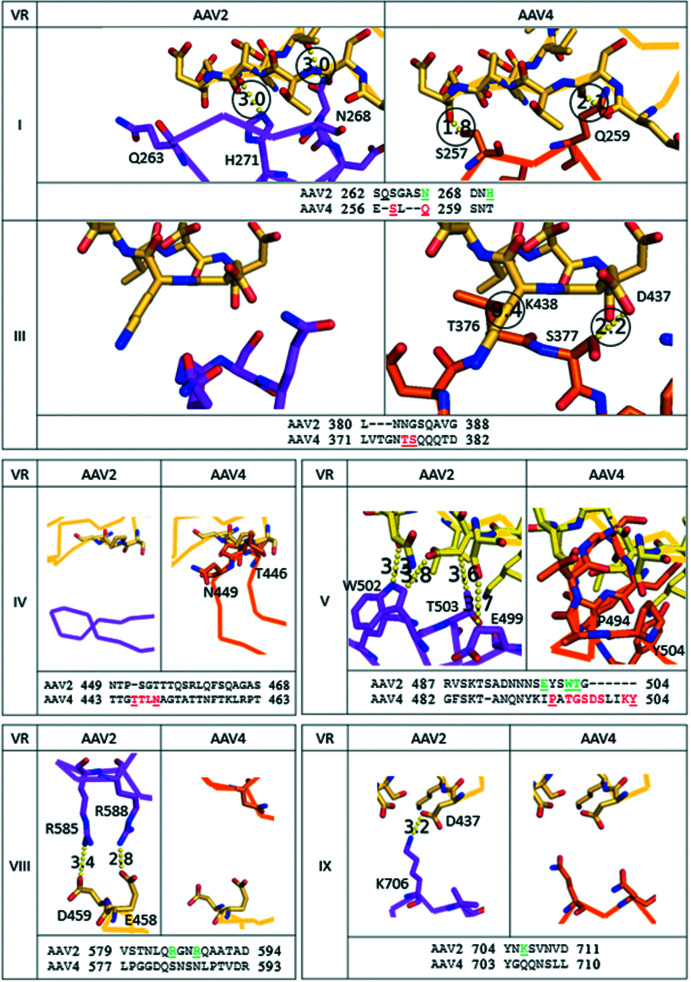
Comparison of AAV2 (purple) and AAV4 (orange) at selected variable regions (VR) where PKD2 (yellow) interacts with AAV2. Amino-acid sequence alignments for each VR are provided below each set of structures, with the locations of the first and last residues provided (according to VP1 numbering); residues of interest extending beyond the variable region are supplied after the number; contact residues are shown in green, while clashing residues are shown in red. Too many clashes are present for AAV4 VR-IV and VR-V to be shown. Residues of interest are labeled on the structure and underlined in the alignment. Distances that are less than or equal to 3.0 Å are surrounded by a circle to make them easier to locate.

VR-III contains four residues that are important for the binding of AAV2 to PKD2. There is considerable variation between AAV2 and some of the other serotypes (Fig. 4 ▸, Supplementary Fig. S8). VR-III of AAV4 clade members (which are AAVR-independent) has a structure that is distinct from that shared by the majority of AAVs that, like AAV2, are PKD2 binders (Supplementary Fig. S8). Within the AAV4 clade, AAV4 is the outlier in sequence and structure, but AAV4 clade members are much more like each other than other AAVs. Steric conflict would be substantial between AAVR Lys438 and AAV4 Thr376. Conflict at the backbone of AAVR Asp437 is somewhat less but significant, and there is also conflict predicted with a conserved asparagine in AAV11, AAV12 and AAVrh32.33. AAV5, like AAV4, does not bind AAVR PKD2. Although AAV5 Asp374 and Thr376 are translated ∼2 Å relative to AAV4, this same region of VR-III would clash with PKD2 near Lys438 in both AAV5 and the AAV4 clade (Supplementary Fig. S8).

VR-I is immediately adjacent to VR-III in the 3D structure. In AAV2, six of the residues that interact with PKD2 come from VR-I, with one additional residue (His271) immediately downstream. As shown in Fig. 4 ▸, VR-I in AAV4 (and most other serotypes; Supplementary Fig. S9) shows important differences from that seen in AAV2. Surprisingly, steric clashes are seen in AAV7, AAV8 and AAV9 (all of which are shown, or implied, to rely upon AAVR for entry into the cell; Dudek *et al.*, 2018 ▸), where AAVR appears to clash with AAV7 and AAV9 at a single residue (Ser268 in both strains) and with multiple residues in AAV8. The PKD1-dependent AAV5 clashes with PKD2 at two residues: Ser254 and Gly257. The AAV4 clade (which are AAVR-independent) clash with PKD2. AAV4 and AAV12 each contain two residues (Ser257 and Gln259 in AAV4 and Thr266 and Asn268 in AAV12) that would clash with PKD2. MD simulations (below) show that this AAV4 clash can be resolved, but its accommodation presumably contributes to the less favorable binding energy. AAV11 and AAVrh32.33 have a single residue (Thr257) that would conflict with PKD2 (Asp436 and Asp437).

The third region of AAV2 that interacts with PKD2 is VR-V, which contains three residues of interest. A comparison of VR-V between AAV2 and AAV4 is presented (Fig. 4 ▸ or, for additional serotypes, Supplementary Fig. S10). All AAV4 clade members show a substantial clash between VR-V and PKD2 that is unique to the clade. It is noteworthy that although AAV5, like AAV4, does not bind PKD2, the structure of AAV5 VR-V is very similar to those of the AAV2-like serotypes that bind PKD2. Thus, VR-V is unlikely to be the cause of the differences in AAVR binding between AAV5 and AAV2.

The next region of AAV2 that interacts with PKD2 is VR-VI, which contains two AAV2 residues in close contact. VR-VI from various serotypes is compared in Supplementary Fig. S11. The loop in the AAVR-independent AAV4 clade members is further removed from PKD2 and would likely not be part of any interaction.

PKD2 interacts with AAV2 VR-VIII from two symmetry-equivalent subunits. In the first, AAV2 Gln589 contacts AAVR Ser425 (Supplementary Fig. S12, located at the beginning of PKD2 βB), an interaction that is conserved in AAV1 and AAV9 (both dependent upon AAVR; Dudek *et al.*, 2018 ▸) but not in other AAV serotypes. The second subunit makes contact through the positively charged AAV2 Arg588 and Arg585 of the heparin-binding domain (HBD), which interact with the negatively charged AAVR Glu458 and Asp459 in the PKD2 βD–βE loop at distances of 2.8 and 3.4 Å, respectively (Fig. 4 ▸ and Supplementary Fig. S13). Interestingly, neither residue was identified in either report of the experimental AAV2 structure even though the binding residues in AAVR were identified (Meyer *et al.*, 2019 ▸). All serotypes have polar amino acids in this location, but it is only AAV2 that has the two arginines in the HBD capable of a salt-bridge electrostatic attraction. (Each AAV2 capsid has 60 symmetry-equivalent R*XX*R motifs, but at each one the glycan attachment and PKD2-binding interactions would be mutually exclusive; Meyer *et al.*, 2019 ▸). The length of the arginine side chains in AAV2 allows a favorable PKD2 electrostatic interaction without distortion of either the AAV or AAVR structure. Indeed, we do not see systematic differences in the docking of PKD2 between AAV2 and AAV1 (Meyer *et al.*, 2019 ▸; Zhang, Xu *et al.*, 2019 ▸). We divided the VR-VIII interactions according to the two AAV subunits involved (Supplementary Figs. S12 and S13), but neither shows any potential for steric hindrance from any of the superimposed AAV structures. The conclusion is that the favorable influence of the HBD in AAV2 is modest and that there is no other reason to consider VR-VIII to be a major determinant in the mode of AAVR binding.

The final region to be considered with respect to PKD2 is VR-IX (Fig. 4 ▸ and Supplementary Fig. S14). Relevant to the observed 3.2 Å salt bridge between AAV2 Lys706 and AAVR Asp437 (Meyer *et al.*, 2019 ▸), the lysine is conserved in all clades that bind PKD2, but not AAV5 and AAV4. Replacing the lysine is an aspartic acid in AAV5 and one of several polar residues (asparagine, glutamine or threonine) in the AAV4 clade. In summary, we have a potentially favorable electrostatic contribution from VR-IX in all clades known to bind PKD2. This is neutralized in the AAV4 clade with a switch to a polar residue. In AAV5 (which binds PKD1 but not PKD2), the switch to a like (repulsive) charge is in the context of a shorter side chain increasing the distance and mitigating the incompatibility.

Even though VR-II, VR-IV and VR-VII of AAV2 were not seen to interact with PKD2, these regions were analyzed in case any were sufficiently different in AAV4 to account for the lack of binding between AAV4 and PKD2. The contact residue Arg471 is located upstream of VR-IV. A comparison between serotypes for this region is presented in Supplementary Fig. S15. The VR-IV loop in the AAV4 clade has a conformation that is different from other clades; furthermore, residues Thr446–Asn449 in AAV4 are incompatible with the binding of PKD2.

#### 3.4.2. Comparison of AAV4 with the PKD1 binding site as seen in the AAV5 complex

By contrast to clade B and most other serotypes, the interactions of AAV5 with AAVR are mediated exclusively through PKD1 (Pillay *et al.*, 2017 ▸; Dudek *et al.*, 2018 ▸). Even though PKD1 plays an accessory role in the transduction of many serotypes (Pillay *et al.*, 2017 ▸), it had only been observed, at the time of our analysis, in the structures of AAV5 complexes (Zhang, Xu *et al.*, 2019 ▸; Silveria *et al.*, 2020 ▸), to which we turn in examining why the AAV4 structure might be incompatible with PKD1 binding. [PKD1 has recently been observed at the corresponding location in a complex with the homologous goat AAV, AAVGo.1 (Large *et al.*, 2022 ▸).] For AAV5, PKD1-interacting residues come from VR-IV, VR-VII and VR-IX (Table 3 ▸, Fig. 1 ▸ *b*). The structures of AAV4 and related clade members were overlaid on the 2.5 Å resolution structure of the AAV5–AAVR complex to rationalize why PKD1 binding is not possible for the AAV4 clade and why the avidity is sharply reduced in other non-AAV5 clades.

**Figure 5:**
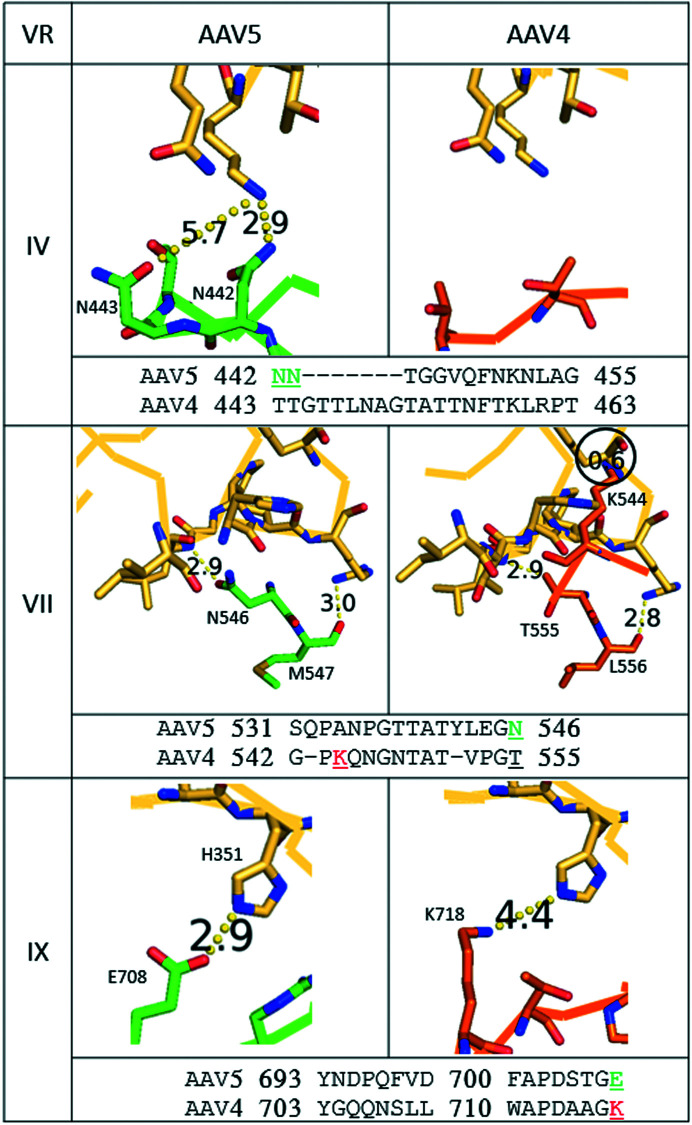
Comparison of AAV5 (green) and AAV4 (orange) at selected variable regions (VR) where PKD1 (yellow) interacts with AAV5. Amino-acid sequence alignments for each VR are provided below the set of structures, with numbers showing the first and last residues of the VR (according to VP1 numbering); residues of interest extending beyond the variable region are supplied after the number; contact residues are shown in green, while clashing residues are shown in red. Residues extending beyond VR-IX are shown in the VR-IX alignment. Residues of interest are labeled on the structures and underlined in the alignment. The clash between AAVR and AAV4 for VR-VII is surrounded by a circle to highlight its presence.

The first region of interest is variable region 4 (VR-IV), with two AAV5 residues contacting PKD1 (Fig. 5 ▸ and Supplementary Fig. S16). VR-IV differs in length between the major branches of the AAV family, with AAV5 having the shortest loop. AAV4-like viruses have the longest loop, but the additional residues extend the loop away from AAVR Lys399. The AAV4-like loop passes Lys399 with small polar residues: there are no unfavorable steric or electrostatic interactions. By contrast, when aligned with the AAV5 complex, an arginine in AAV2 (Arg459) or a lysine in AAV9 (Lys462) would be in close proximity to AAVR Lys399, potentially leading to unfavorable steric and/or electrostatic interactions with PKD1 if it were bound as in AAV5.

Consider now VR-VII. The AAV5 loop is three residues longer than in most serotypes and forms a hairpin structure well separated from PKD1 (Fig. 5 ▸ and Supplementary Fig. S17). In AAV5 this region does not change significantly on binding AAVR (Silveria *et al.*, 2020 ▸). VR-VII differs in the AAV4 clade, with a fold in the native structure that would overlap with bound PKD1. Again, the MD simulation (below) shows flexible accommodation, but at a cost to the binding energy. The most severe would be between AAVR Tyr355 and AAV4 Lys544. For other AAV4 clade members (AAV11, AAV12 and AAVrh32.33), there are extensive clashes as well. Other PKD1-independent clades (for example AAV1, AAV2, AAV7, AAV8 and AAV9), when superimposed on the AAV5 complex, are predicted to have multiple clashes with the PKD1 region of AAVR, Gly375–Leu376 and Ile349–His351. It appears that all serotypes, except those like AAV5, have VR-VII loops that would be completely incompatible with PKD1 binding unless there is an induced conformational change.

Finally, consider VR-IX (Fig. 5 ▸ and Supplementary Fig. S18). The structure of the AAV5 complex shows a favorable electrostatic salt bridge at 2.9 Å between AAVR His351 and AAV5 Glu708, which is not conserved. By contrast, the AAV4 clade members (which do not rely upon PKD1 for infection of cells) have positively charged residues at this location (AAV4 Lys718 and AAV12 His728) that would not interact favorably with AAVR His351. No other clashes were seen in the other AAV serotypes analyzed.

#### 3.4.3. MD simulations, stability and free energies of binding

To this point, the binding (or not) of AAV4 to AAVR has been rationalized through analysis of rigid-body superimposition. Here, MD simulation is used to explore the possibility that flexibility in the protein structures could allow PKD1 or PKD2 complexes to form. Our analysis starts with the AAV9 ‘positive control’. Following docking and MD simulation, the structure (final frame) differed from the experimentally determined PKD2–AAV9 complex (PDB entry 7wjx) by 2.9 Å.

**Figure 6:**
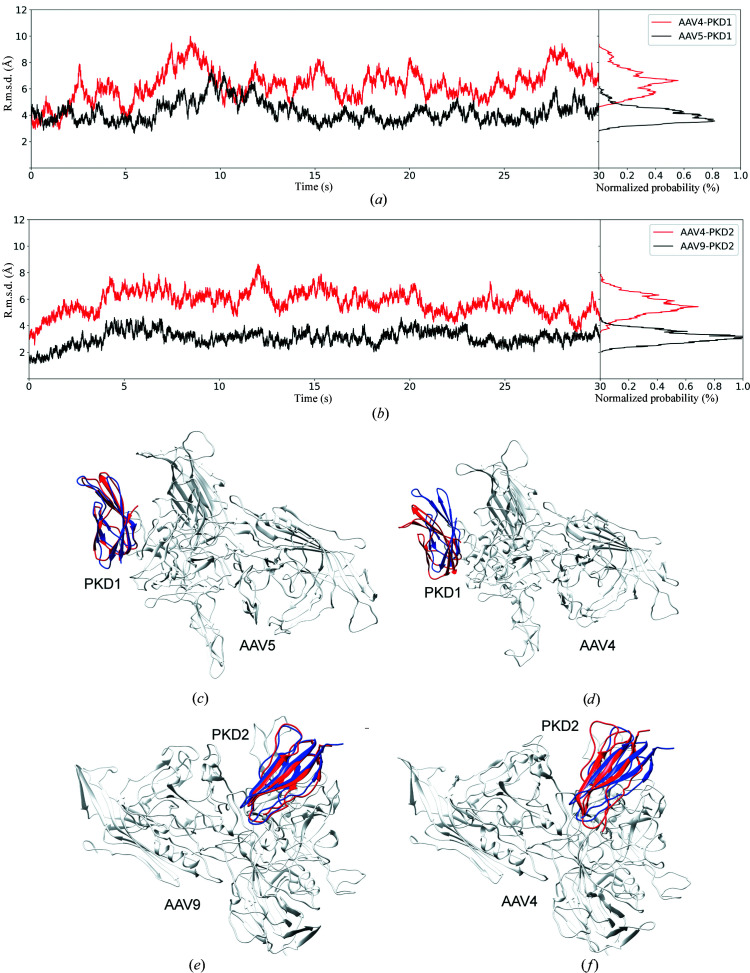
Variation in AAVR PKD1 and PKD2 domain structures during the MD simulations. (*a*) Frame r.m.s.d. of PKD1 in complex with the AAV5 dimer (black) and the hypothetical AAV4 dimer (red). (*b*) PKD2 in complex with the AAV9 dimer (black) and the hypothetical AAV4 dimer (red). The insets to the right of each graph show the distribution of r.m.s.d. values over the last 15 ns of simulation. (*c*–*f*) Comparison of the initial PKD structure (blue) with the final MD snapshot (red). AAVs of the initial structures are shown in white.

Variation in the receptor structure is tracked during the MD simulation in Fig. 6 ▸. The median backbone r.m.s.d. for PKD1 in a hypothetical complex with AAV4 is 6.5 Å, compared with 3.5 Å for the AAV5 complex that we know to exist. For PKD2 the median r.m.s.d. is 5.5 Å, compared with 3.0 Å for the AAV9 complex that is stable. Larger changes are also observed in the AAV4 hypothetical complexes, comparing the starting and ending structures of MD, than in the AAV5 and AAV9 complexes which are known to exist. However, the wider variation of PKD1 and PKD2 in AAV4 complexes suggests that these complexes are less stable than those of other AAVs that exist in nature. A similar picture emerges from analysis of the virus side of the interface. The *B* factors calculated from the displacements of interface loop residues during MD are predominantly higher for the predicted AAV4 complexes than for those of AAV5 or AAV9.

The free energies of binding for each complex, estimated using MM/PBSA (Kollman *et al.*, 2000 ▸; Massova & Kollman, 2000 ▸), are analyzed in Table 4 ▸. It shows that the relative free energy of binding (ΔΔ*G*) between AAV4–PKD1 and AAV5–PKD1 was 29.6 kcal mol^−1^, indicating that PKD1 binds much more strongly to AAV5 than it would to AAV4. Similarly, PKD2 binds much more tightly to AAV9 than it is predicted to bind to AAV4, as the ΔΔ*G* between these two complexes was 11.4 kcal mol^−1^. Notwithstanding the intractability of performing simulations for long enough to see the full dissociation of AAVR–AAV4 complexes, the free-energy calculations show much weaker binding for the hypothetical AAV4–PKD12 complexes than for AAV5 or AAV9, even under conditions in which the structures have the flexibility to adapt to more favorable configurations. This is consistent with the experimental findings that AAV4 does not interact with PKD12.

## 4. Conclusions

In this article, we report a high-resolution structure of AAV4 at 2.2 Å resolution. We then compared it with AAV structures known to interact with AAVR to suggest reasons why AAV4 might not be able to form a complex with the receptor, which is necessary for all other AAV serotypes to infect cells. We specifically considered the PKD2 and PKD1 portions of AAVR that are known to interact with AAV2 and AAV5, respectively.

Multiple differences were identified between AAV4 and AAV2 which may explain why binding might not be possible between AAV4 and PKD2 (see Supplementary Fig. S19 for a summary). Many of the residues identified as contact residues in AAV2 are different in the AAV4 clade. Regions where the differences might be of highest impact include VR-I, VR-III, VR-IV and VR-V, which result in multiple steric clashes for a potential interaction with PKD2. AAV5, like AAV4, is incompatible with PKD2. AAV5 VR-I and VR-III would be in conflict with AAV2-like binding of PKD2, but AAV5 is more similar to PKD2-binding serotypes in other loops of the binding site of AAV2. MD simulations indicated that AAV4 does not adapt for favorable interactions with AAVR at either of the sites that would correspond to the PKD2 site for AAV2 and most other AAVs, or the PKD1 site in the AAV5-like clade.

In terms of understanding why AAV4 and other clades do not bind PKD1 like AAV5, the answer appears to be simpler (Supplementary Fig. S20). It is AAV5 alone that has a VR-VII conformation that is compatible with binding. It is possible that differences in VR-IX could have a secondary role. We see that a favorable salt bridge in the AAV5 complex is lost in other clades, and a potentially unfavorable electrostatic environment for AAVR His351 in the AAV4 clade.

Despite sharing 30% identity, the PKD1 and PKD2 domains of AAVR interact with AAV in different ways. Other PKD domains (PKD3 through PKD5) are slightly more similar (33–38% identity to PKD2) but do not interact directly with AAV. Evolutionarily related viruses using the same receptor typically interact in the same way, so the different modes of interaction among AAVs with different AAVR domains bound at distinct sites on different AAV serotypes are quite unique (Zhang, Xu *et al.*, 2019 ▸). The results reported here explain, in molecular detail, why AAV4 differs from both AAV2-like and AAV5-like viruses and does not bind AAVR at either PKD1 or PKD2. It is not yet known what the functional replacement of AAVR in AAV4 infection is and how it might interact.

With ongoing interest in AAV as the delivery vector of gene-therapy treatments, research into understanding the mechanisms of virus–receptor interactions is an essential foundation for improving the efficiency and specificity of gene delivery. The structure of AAV4 presented here, and the subsequent comparative analysis using receptor complexes of homologous strains, has allowed a narrowing down of the viral determinants of the modes of binding to the predominant cellular receptor. There appears to be only one conformation of surface loop VR-VII that is compatible with AAV binding to the PKD1 domain of AAVR, like AAV5. For AAV2-like binding to the PKD2 domain of AAVR, several loops need to be configured appropriately: VR-I, VR-III, VR-IV and VR-V. These are located in two regions of the viral surface: VR-III and VR-I are on the shoulder extending down from each spike towards the twofold axis and VR-IV and VR-V are further towards the top of the spikes (Fig. 3 ▸). These two regions interact with the N-terminal and C-terminal ends of the PKD2 domain, respectively, as tightly bound in the AAV2 complex (Fig. 1 ▸).

## 5. Related literature

The following references are cited in the supporting information for this article: Bennett *et al.* (2017 ▸), Pacouret *et al.* (2017 ▸) and Rieser *et al.* (2020 ▸).

## Supplementary Material

PDB reference: adeno-associated virus serotype 4, 7thr

EMDB reference: adeno-associated virus serotype 4, EMD-25903

Supplementary Tables and Figures. DOI: 10.1107/S2059798322012190/ni5023sup1.pdf

## Figures and Tables

**Table 1:** Cryo-EM data collection, processing and refinement of the AAV4 structure

Data collection	
Magnification	165000×
Voltage (V)	300
Electron exposure (e^−^ Å^−2^)	47
Defocus range	−3.9 to 1.0
Pixel size (pre-refinement) (Å)	0.253
Pixel size (post-refinement) (Å)	0.254
Data processing	
Motion correction	*RELION* 3.1.1
CTF estimation	*CTFFIND*4.1
Symmetry imposed	*I*1
Initial particle images	85449
Final particle images	85411
Map resolution (Å)	2.21
FSC threshold	0.143
Refinement	
Protein atoms per asymmetric unit (mean *B* factor, Å^2^)	4333 (21.9)
Solvation	219 H_2_O/2 Mg^2+^
R.m.s.d. from ideal, bond lengths (Å)	0.11
R.m.s.d. from ideal, bond angles (°)	1.3
Ramachandran outliers	1
Cross-correlation (model–map; grid points within 2.2 Å of atoms)	0.871
Envelope correction/equivalent overall *B* factor (for optimal fitting of model to sharpened map) (Å^2^)	−4.3/−17.2
Resolution from model–map refinement (*RSRef* *d*_0.5_)	2.3

**Table 2:** Residues on the surface of AAV4 that align with the residues of AAV2 that contact AAVR PKD2 (Meyer *et al.*, 2019 ▸)

VR	AAV2 residue	AAV4 equivalent
I	Gln263	Ser257-Leu258
	Ser264	Ser257-Leu258
	Gly265	Ser257-Leu258
	Ala266	Ser257-Leu258
	Ser267	Ser257-Leu258
	Asn268	Gln259
N/A	His271	Thr262
III	Asn382	Thr373-Gly374-Asn375-Thr376
	Gly383	Thr373-Gly374-Asn375-Thr376
	Ser384	Gln378
	Gln385	Gln379
N/A	Arg471	Ser466
V	Glu499	Ile493
	Trp502	Leu501-Ile502
	Thr503	Lys503
VI	Asp528	Gly527-Pro528
	Asp529	Gly527-Pro528
VIII	Gln589	Leu588
IX	Lys706	Glu705
	Val708	Asn707

**Table 3:** List of contact residues of AAV5 that interact with AAVR (Silveria *et al.*, 2020 ▸) and the corresponding residues of AAV4

VR	AAV5 residue	Equivalent in AAV4	
IV	Asn442	N/A	
	Asn443		
VII	Ser531	Pro543	
	Gln532	Lys544	
	Glu544	Pro553	
	Gly545	Gly554	
	Asn546	Thr555	
	Met547	Leu556	
IX	Gln697	Asn707	
	Phe698	Ser708	
	Glu708	Lys718	
	Arg710	Thr720	
	Thr712	Pro722	

**Table 4:** Free energies of binding for the four PKD12–AAV complexes calculated with MM/PBSA

	Δ*H* (kcal mol^−1^)	−*T*Δ*S* (kcal mol^−1^)	Δ*G* (kcal mol^−1^)
AAV5–PKD1	−52.79	27.62	−25.17
AAV4–PKD1	−22.35	26.77	4.42
ΔΔ*G*			29.59
AAV9–PKD2	−38.08	31.50	−6.58
AAV4–PKD2	−32.08	36.92	4.84
ΔΔ*G*			11.42
