# Supplementary material for: Cryo-EM structure of adeno-associated virus 4 at 2.2 Å resolution
Source: Acta Crystallogr D Struct Biol. 2023 Jan 20;79(Pt 2):140–53. doi: 10.1107/S2059798322012190 (PMC9912921; doi:10.1107/S2059798322012190)
Supplement: Supplementary file 1 [file d-79-00140-sup1.pdf]

STRUCTURAL BIOLOGY

Volume 79 (2023)

Supporting information for article:

Cryo-EM structure of adeno-associated virus 4 at 2.2 Å resolution

Grant Zane, Mark Silveria, Nancy Meyer, Tommi White, Rui Duan, Xiaoqin Zou and Michael Chapman

Table S1 Primers used for cloning of AAV4 into pFastBac-LIC(4A).

Name	Sequence[a,b]	Purpose
GMZ-1b	GACTAGTGAGCTCGTCGACGTAGG	Amplification of pFastBac-LIC(4A); reverse primer
GMZ-2	CACTGATTAAGCATTGGTAACTGTCAGACC	Amplification of pFastBac-LIC(4A); forward primer
GMZ-3	GGTCTGACAGTTACCAATGCTTAATCAGTG	Amplification of pFastBac-LIC(4A); reverse primer
GMZ-4	GTACCAAGCTTGTCGAGAAGTACTAGAGGATC	Amplification of pFastBac-LIC(4A); forward primer
GMZ-7b	CCTACGTCGACGAGCTCACTAGTC<br>AcGACTGACGGTTACCTTCCAGATTG	Amplification of the AAV4-VP1 sequence; forward primer
GMZ-8	GATCCTCTAGTACTTCTCGACAAGCTTGGTAC<br>TTACAGGTGGTGGGTGAGGTAGC	Amplification of the AAV4-VP1 sequence; reverse primer
GMZ-9	CGTATACTCCGGACTATTAATAGATCATGGAG	Sequencing primer
GMZ-10	AACCTCTACAAATGTGGTATGGCTG	Sequencing primer
EEL23	CAGTGAGATGCGTGCAGCAG	Sequencing primer
EEL24	ATGCCTTCTACTGCCTGGAG	Sequencing primer
EEL25	TGACCAGAGCAACAGCAACC	Sequencing primer
EEL27	GAAGCCCTGCTGCTTGATTG	Sequencing primer
EEL28	GCCCAAACCCACCAATCAGC	Sequencing primer

a – underlined sequence reflects the overhang sequence shared with vector sequence.

b – double underlined and lowercase base shows location of mutation within VP1 start codon.

Table S2 Melting temperatures of isolated VLPs for various serotypes and the values published by others to show range of reported values.

Serotype	Average T_m ± standard deviation (this paper)	Rieser *et al.* (2020)	Pacouret *et al.* (2017)	Bennett *et al.* (2017)
AAV2	73.4°C ± 0.13	68°C	69.6°C	77.5°C
AAV5	89.9°C ± 0.06	90°C	88.7°C	89.7°C
AAV4	79.9°C ± 0.03	-	-	76.0°C

Table S3 Average root mean-square deviation (RMSD) values for the side-chain, back-bone and alpha carbon (C_α) for the variable regions (VR) and observed structure for 3.2 Å AAV4 structure (PDBid: 2g8g) compared with the current 2.2 Å structure.

**Region**	**Residues**	**Side-chain**	**Backbone**	**C_α**
VR-I	253-261	0.7	0.6	0.3
VR-II	317-322	1.6	1.1	1.2
VR-III	371-382	0.8	0.4	0.4
VR-IV	443-464	0.9	0.8	0.5
VR-V	482-505	0.7	0.6	0.4
VR-VI	525-541	0.7	0.6	0.5
VR-VII	543-555	0.9	0.7	0.5
VR-VIII	578-593	0.7	0.3	0.3
VR-IX	703-710	0.7	0.4	0.4
All	214-734	0.7	0.5	0.4

Table S4 Largest side chain differences between the current 2.2 Å cryo-EM structure and the prior 3.2 Å crystal structure (PDBid: 2g8g).

Listed are three residues that would clash with AAVR as well as the top 25 residues by root mean-square difference (RMSD) after least-squares superposition. It is possible that differences result due to differing interpretation in ambiguous regions of the map for one or other, but this is difficult to appraise in the absence of structure factors or experimental maps for PDBid 2g8g that were never deposited. Reported B-factors can serve as a proxy. They indicate that VR-II is somewhat disordered in both structures. However, two thirds of differences are at sites where the 2.2 Å resolution map defines the conformation unambiguously.

Residue	VR	RMSD (Å)	$\langle B_{3.2} \rangle$ (Å^2)	$\langle B_{2.2} \rangle$ (Å^2)	Observations, 2.2 Å map	Comment
D225		1.9	29.9	40.0	Clear sidechain	
E230		3.2	49.8	41.4	Ambiguous sidechain	
V242		1.5	15.5	8.2	Clear sidechain	
Q259	I	1.3	41.7	33.5	Clear sidechain	Both clash with PKD2
K305		2.0	29.2	14.5	Clear sidechain	
S317	II	1.6	88.6	73.0	Ambiguous sidechain	
N318	II	1.6	94.0	76.8	Ambiguous sidechain	
G319	II	1.7	86.4	75.2	Ambiguous sidechain	
E320	II	2.3	82.3	72.8	Ambiguous sidechain	
T321	II	1.5	66.8	63.6	Ambiguous sidechain	
E340		3.5	48.3	19.1	Clear sidechain	
T376	III	1.3	38.3	34.2	Clear sidechain	Both clash with PKD2
T399		1.8	26.7	19.4	Clear sidechain	
E410		1.5	35.0	18.7	Clear sidechain	
T447	IV	1.2	49.1	47.9	Ambiguous sidechain	Both clash with PKD1
T454	IV	1.5	48.7	40.5	Clear sidechain	
T518		2.3	42.9	26.2	Clear sidechain	
N535	VI	1.5	38.2	29.6	Clear sidechain	
R593	VIII	1.5	53.6	44.7	Ambiguous sidechain	
I610		1.5	22.0	12.4	Clear sidechain	
K639		2.9	30.2	22.5	Clear sidechain	
N666		1.4	39.8	30.4	Clear sidechain	
V679		1.5	20.8	8.8	Clear sidechain	
K687		2.1	41.6	26.6	Clear sidechain	
E688		1.5	49.5	23.3	Clear sidechain	
R689		1.7	60.4	33.6	Clear sidechain	
S701		1.5	33.9	21.5	Clear sidechain	
T731		1.5	26.7	13.1	Clear sidechain	

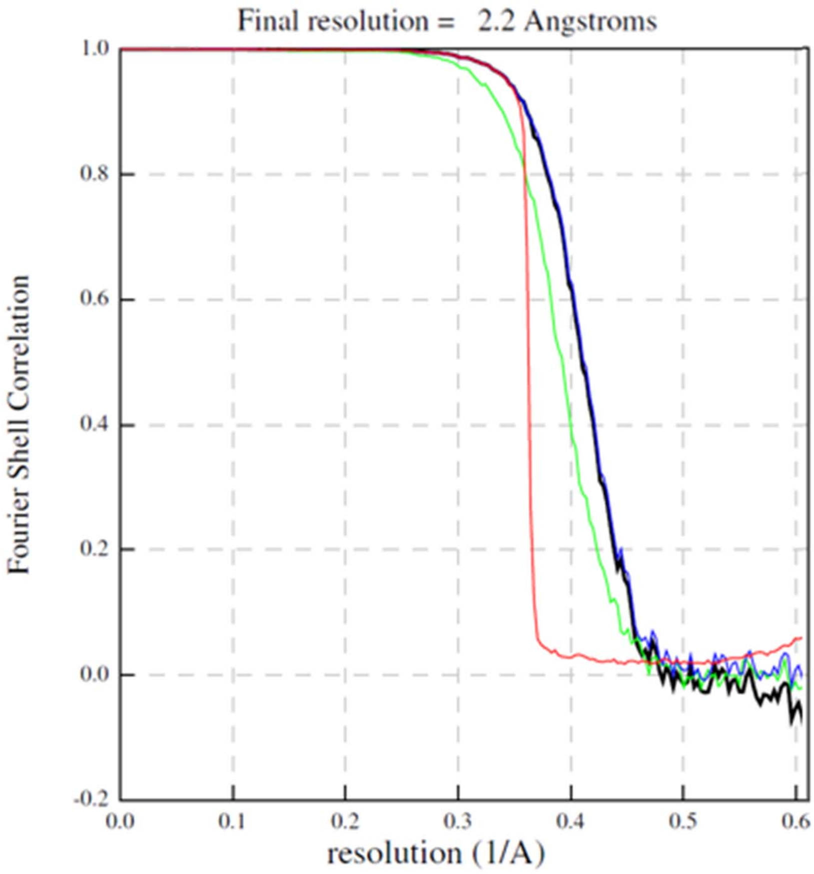

Figure S1 FSC curve for the 2.2 Å AAV4 structure reported in this study. The final resolution was determined at FSC of 0.143. Black line shows corrected Fourier shell correlation; green line shows unmasked Fourier shell correlation; blue line shows masked Fourier shell correlation; and red line shows phase randomized masked Fourier shell correlation.

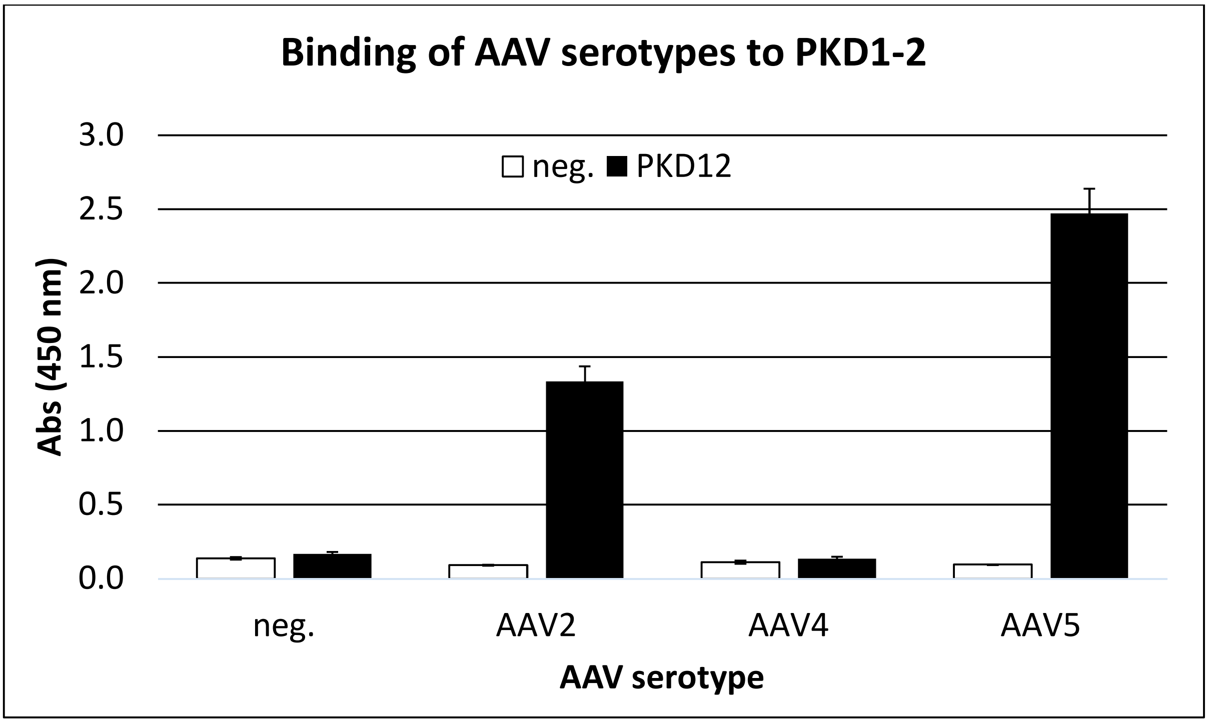

Figure S2 ELISA measurement of PKD12 binding avidity to AAV2, AAV4 and AAV5. Negative control (virus alone, no added ligand; unshaded bars) compared with PKD12 bound samples (shaded). Values are the averages of three replicates with the error bar representing the standard deviation.

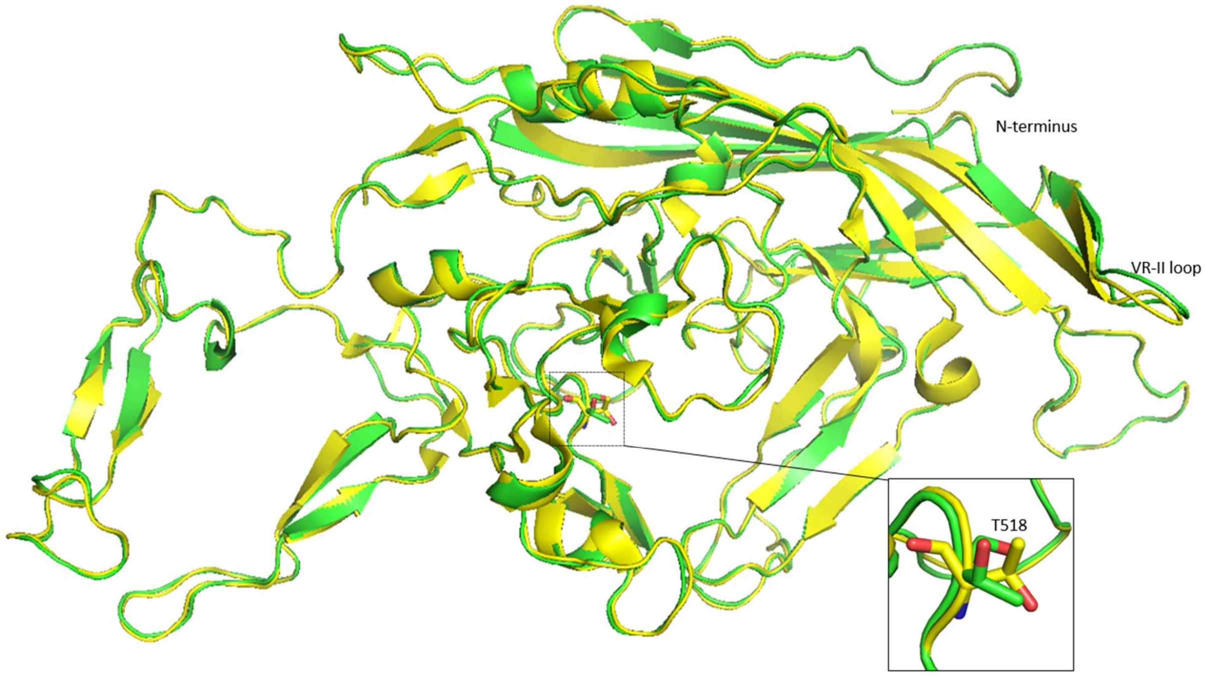

Figure S3 Superimposition of the 3.2 Å structure (PDBid: 2g8g, yellow) with the 2.2 Å structure (green). The β-strands at the top of the figure line the inner surface of the capsid, while the loops at the bottom decorate the outer surface of AAV. The 3.2 Å structure extends 4 residues beyond the N-terminus of the 2.2 Å structure (top right). The EM map for the VR-II loop (labeled) is somewhat disordered, and both structures have high B-factors here. The residue with the highest back-bone RMS difference (2.3Å), T518, is enlarged.

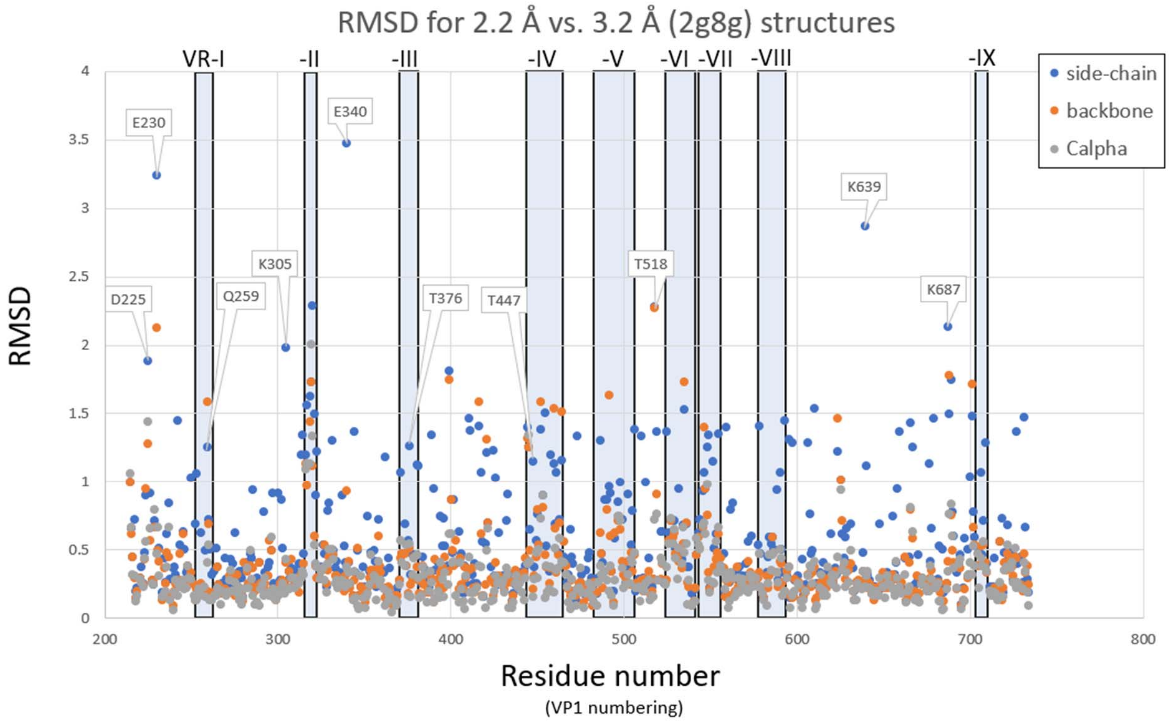

Figure S4 Coordinate differences between the 3.2 Å structure (PDBid: 2g8g) and the 2.2 Å structure reported here, shown as per-residue root mean square deviations (RMSD).

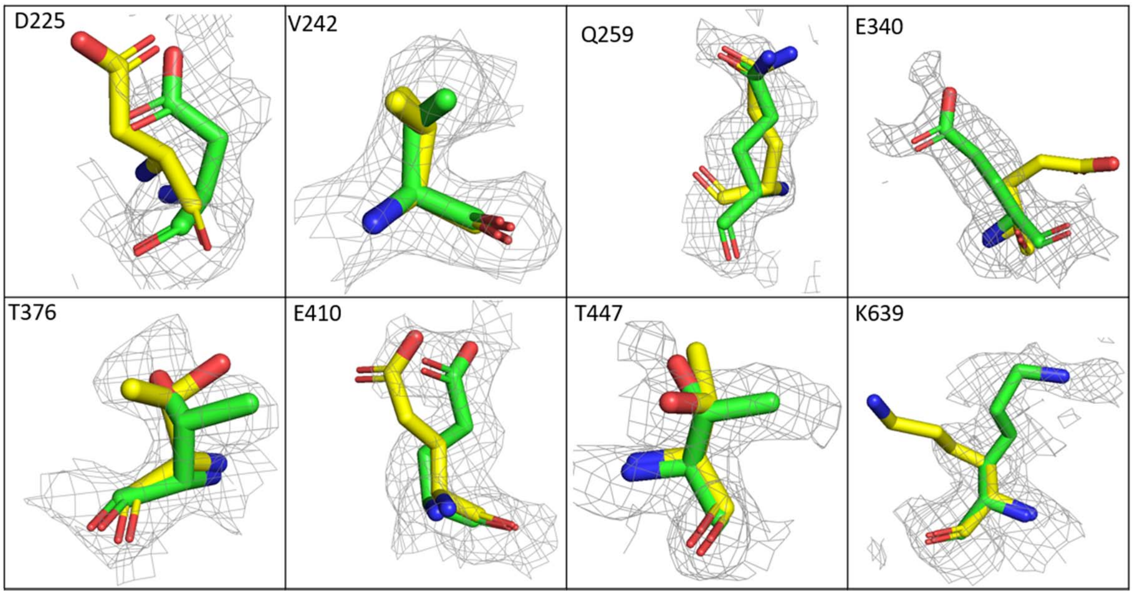

Figure S5 Comparison of select residues from 2.2 Å structure (green) and 3.2 Å structure (PDBid: 2g8g; yellow). Coulombic potential from the current work is contoured at 1.0 σ.

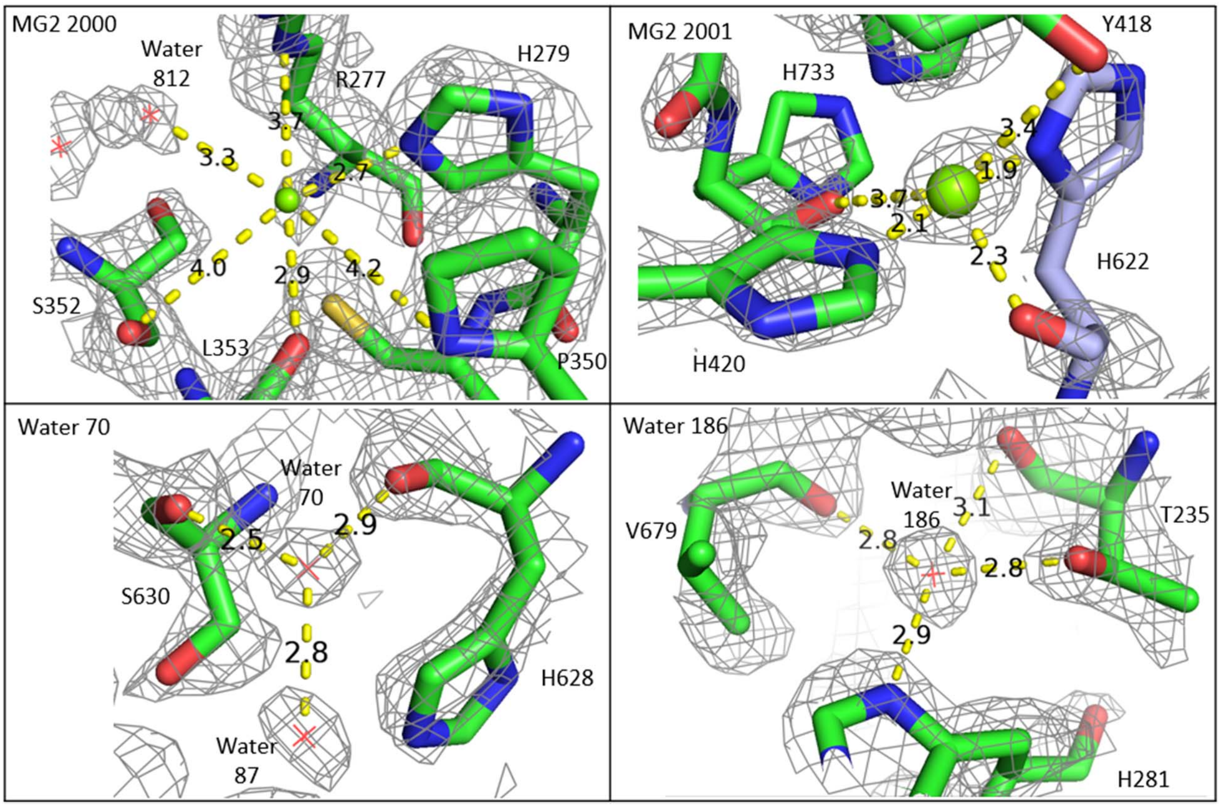

Figure S6 Location of magnesium ions (green sphere) and example water molecules (pink crosses) from 2.2 Å AAV4 structure (green carbons). The coulombic potential (grey wire-frame) is contoured at 3.0 σ. Atoms from neighboring subunits are shown with light blue carbons.

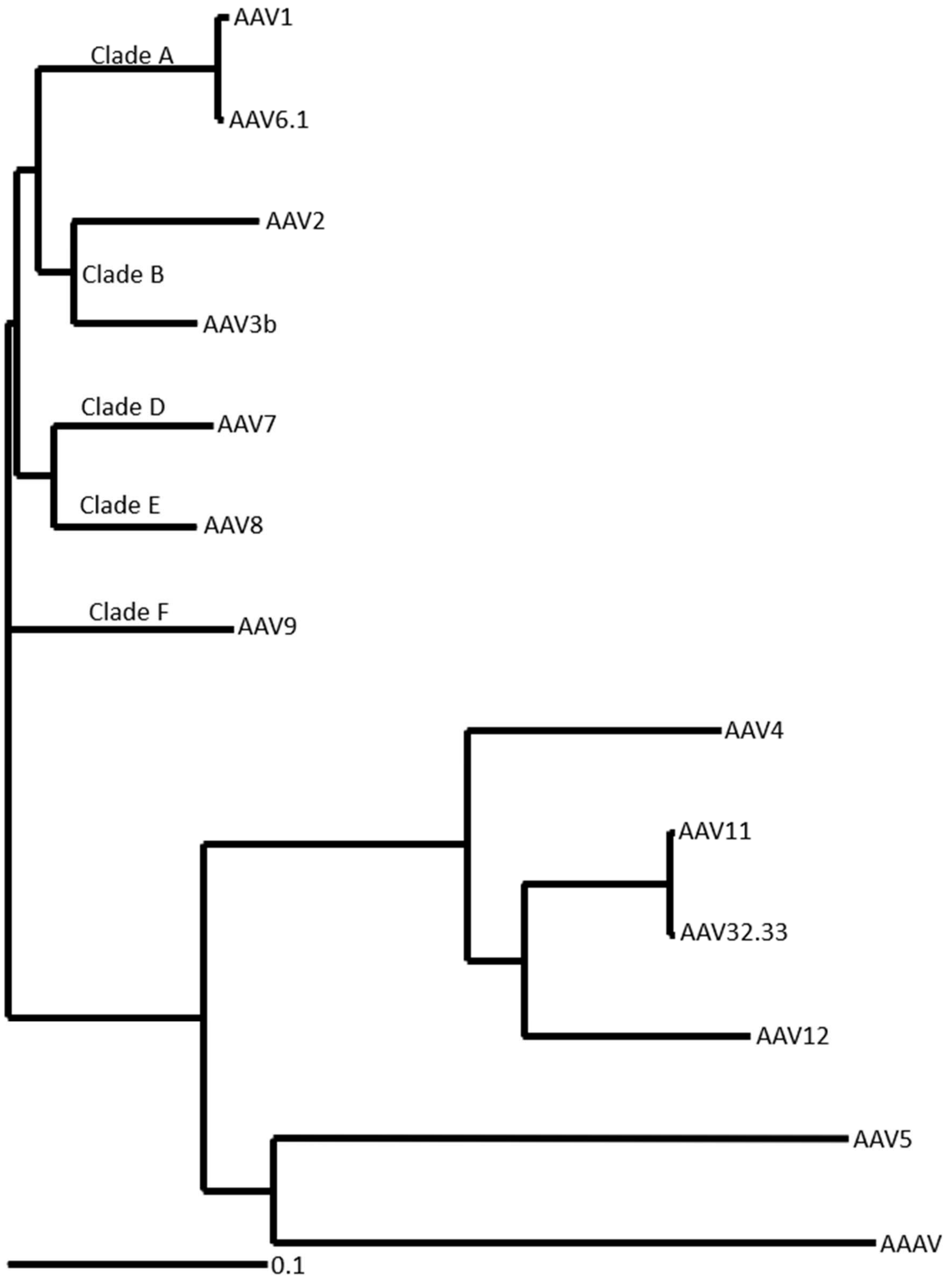

Figure S7 AAV Phylogenetic tree. Representative primate AAV serotypes, discussed in this paper are included as well as avian AAV (AAAV). The scale bar shows the fraction of sites with substitutions.

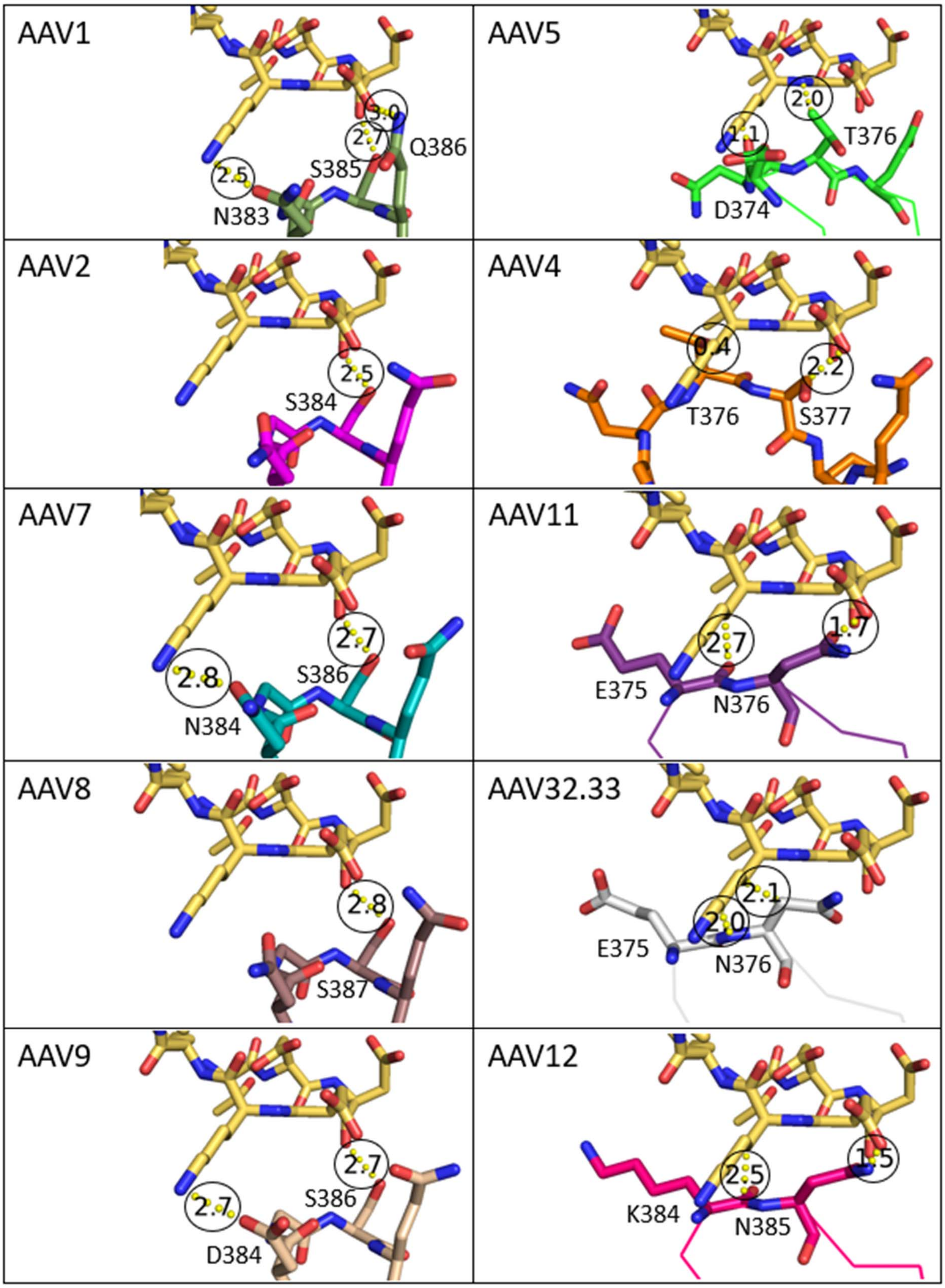
AAV1
3.0
2.7
Q386
S385
2.5
N383
AAV5
2.0
1.1
T376
D374
AAV2
2.5
S384
AAV4
0.4
2.2
T376
S377
AAV7
2.7
2.8
S386
N384
AAV11
2.7
1.7
E375
N376
AAV8
2.8
S387
AAV32.33
2.1
2.0
E375
N376
AAV9
2.7
2.7
S386
D384
AAV12
1.5
2.5
K384
N385

Figure S8 Potential interactions of variable region 3 (VR-III) for AAV structures after alignment to the AAV2-PKD12 receptor complex (PDBid: 6nz0). Carbons are colored yellow for AAVR and by serotype for AAV structures. The following structures were used: AAV1 (dark green; PDBid: 6jcq), AAV2 (magenta; PDBid: 6nz0), AAV4 (orange; PDBid: 7thr), AAV5 (green; PDBid: 7kpn), AAV7 (cyan; PDBid: 7l5q), AAV8 (brown; PDBid: 2qa0), AAV9 (wheat; PDBid: 3ux1), AAV11 (purple; PDBid: 7l6f), AAV12 (hot pink; PDBid: 7l6b), and AAVrh32.33 (grey; PDBid: 4iov). Steric clashes (circled) would occur between AAVR-K438 and AAV4-T376 (or AAV5-D374 and -T376 or AAV11-N376, 12-N385, rh32.33-N376) and also between the side-chains of AAVR-D437 and AAV4-S377 (or AAV11-N376 and AAV12-N385).

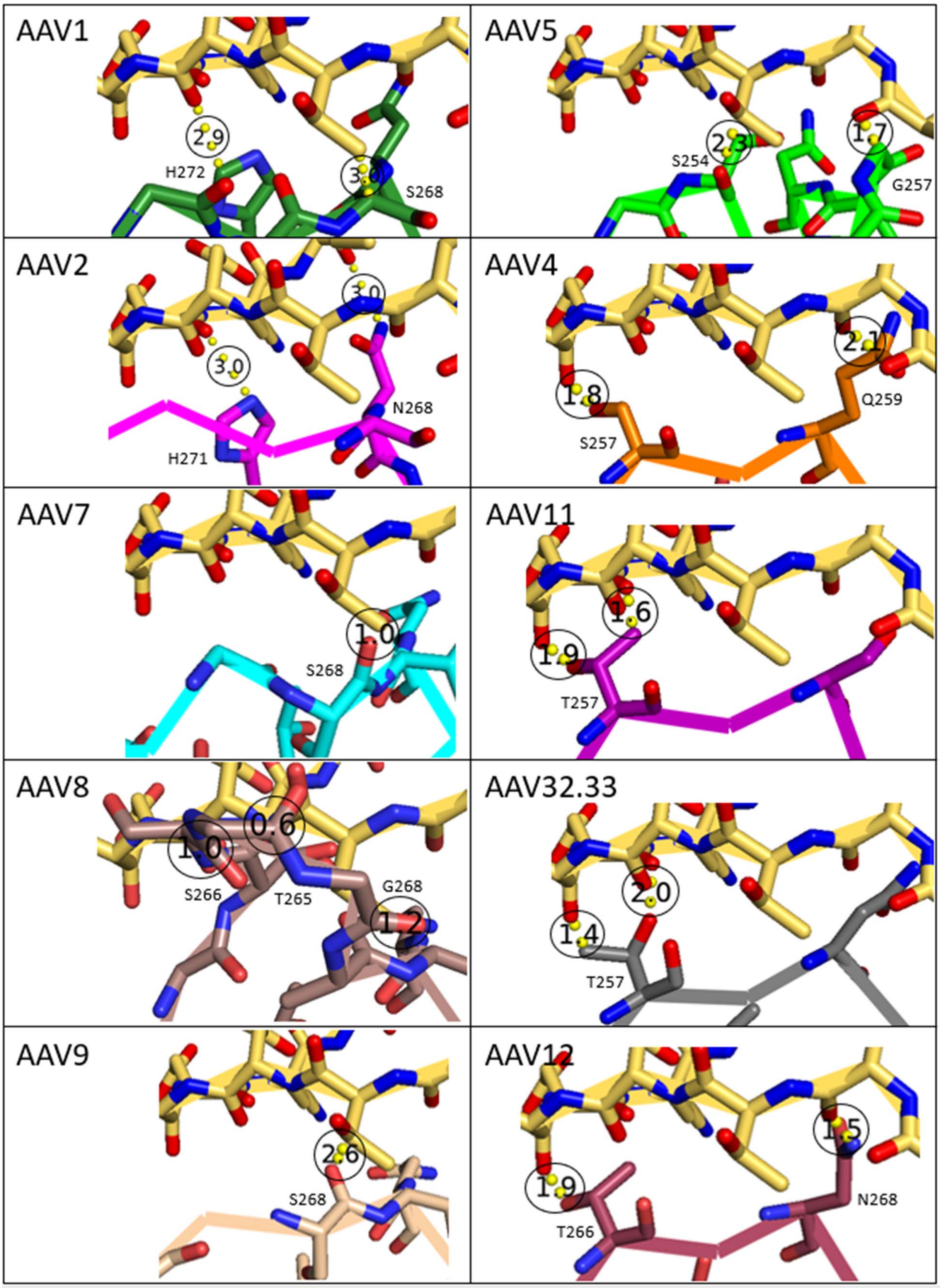
AAV1
2.9
H272
3.0
S268
AAV5
2.3
S254
1.7
G257
AAV2
3.0
3.0
N268
H271
AAV4
2.1
1.8
Q259
S257
AAV7
1.0
S268
AAV11
1.6
1.9
T257
AAV8
0.6
1.0
S266
T265
G268
1.2
AAV32.33
2.0
1.4
T257
AAV9
2.6
S268
AAV12
1.5
1.9
N268
T266

Figure S9 Potential interactions of variable region 1 (VR-I) for AAV structures after alignment to the AAV2-PKD12 receptor complex (PDBid: 6nz0). Structures and serotype color-coding are as described in Error! Reference source not found., and AAVR is shown with yellow carbons. There are multiple clashes between PKD2 and most of the AAV serotypes, including the AAV4-clade and AAV5. Surprisingly, there appear to be clashes in several AAVR-dependent strains (AAV7, 8, 9), which are known empirically to interact with AAVR, suggesting that conformational adaptation may occur with some serotypes.

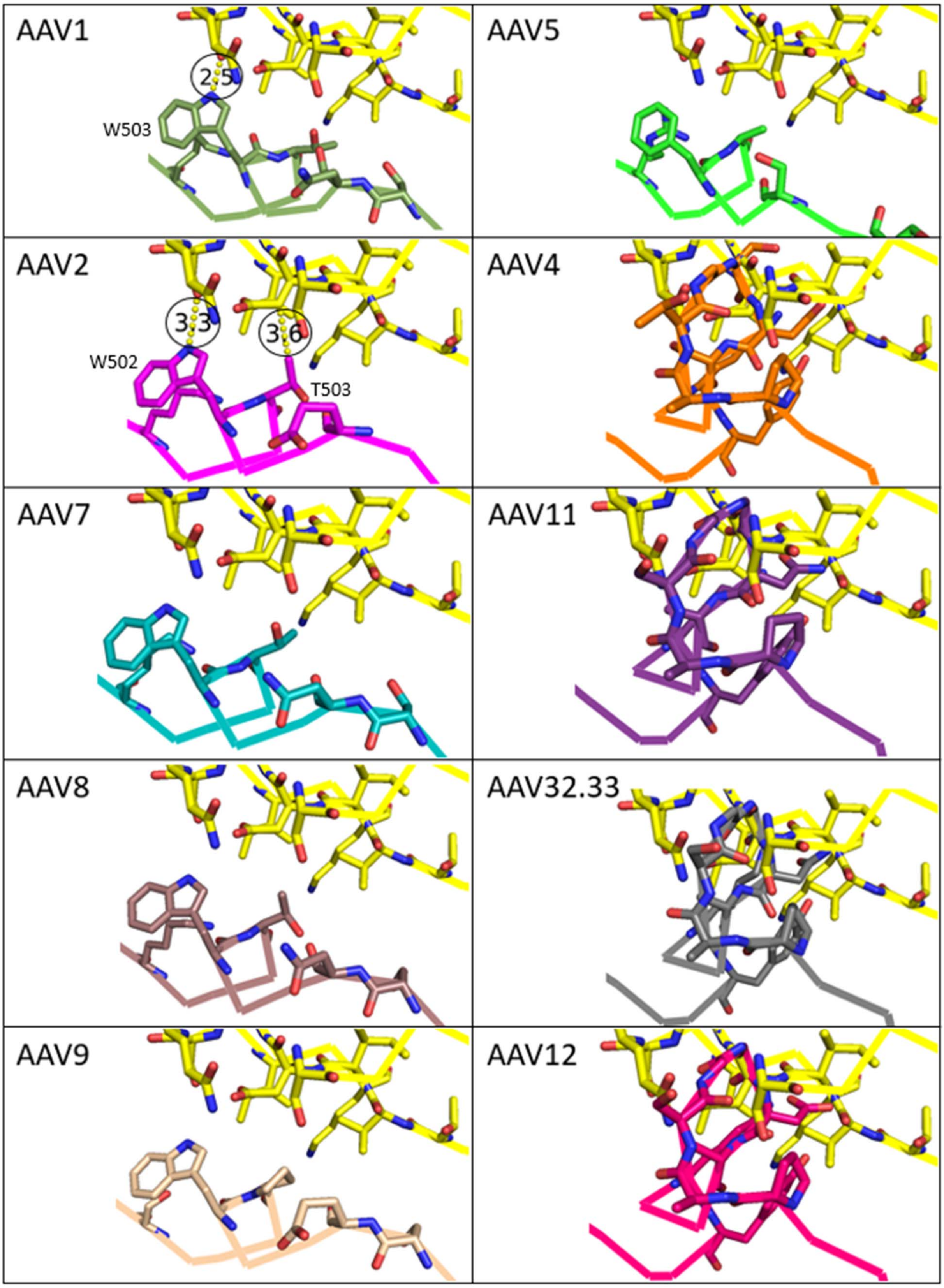
AAV1
2.5
W503
AAV5
AAV2
3.3
3.6
W502
T503
AAV4
AAV7
AAV11
AAV8
AAV32.33
AAV9
AAV12

Figure S10 Potential interactions of variable region 5 (VR-V) for modelled AAV-AAVR complexes predicted by alignment to AAV2-PKD12 (PDBid: 6nz0). Structures and serotype color-coding are as described in Error! Reference source not found., and AAVR is shown with yellow carbons. There would be considerable steric clashes between PKD2 and all AAV4-clade members for residues P494- Y504 in AAV4 and corresponding residues in other clade members. There are no clashes for known PKD2-binders like AAV2, but also no clashes for PKD2-independent AAV5.

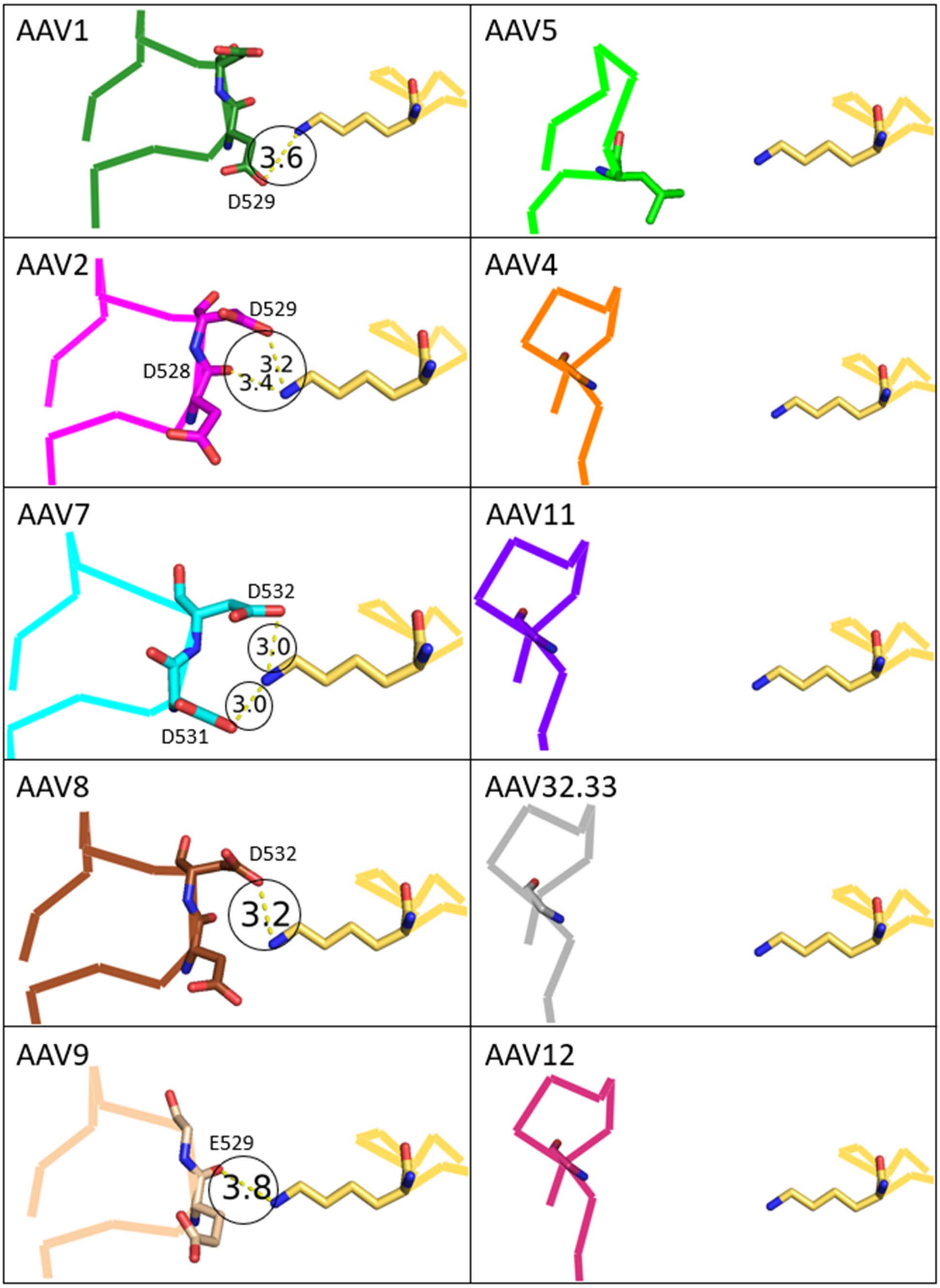
AAV1
3.6
D529
AAV5
AAV2
D529
3.2
D528
3.4
AAV4
AAV7
D532
3.0
3.0
D531
AAV11
AAV8
D532
3.2
AAV32.33
AAV9
E529
3.8
AAV12

Figure S11 Potential interactions of variable region 6 (VR-VI) for AAV structures after alignment to the AAV2-PKD12 receptor complex (PDBid: 6nz0). Structures and serotype color-coding are as described in Error! Reference source not found., and AAVR is shown with yellow carbons. No steric hindrance was observed for this region between PKD2 and these serotypes. Residues in AAV5 and the AAV4-clade that correspond to contact residues in AAV2 are not as close to AAVR. Additionally, an aspartic acid (AAV2-D529 which is in contact with AAVR-K438) is conserved in clades A-E but is a glycine (G530) in AAV9.

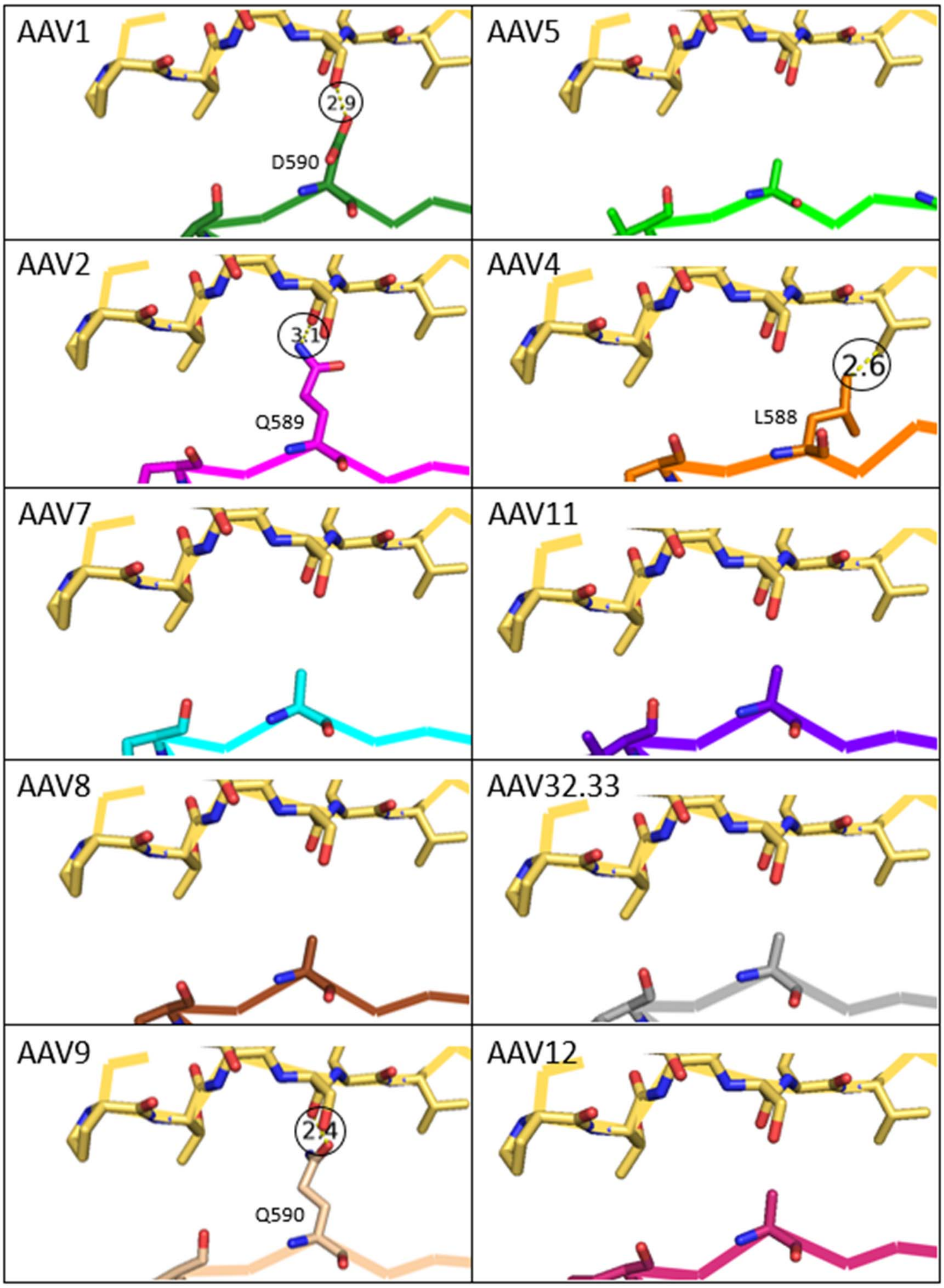

Figure S12 Potential interactions of variable region 8 (VR-VIII) for AAV structures after alignment to the AAV2-PKD12 receptor complex (PDBid: 6nz0). Structures and serotype color-coding are as described in Error! Reference source not found., and AAVR is shown with yellow carbons. No steric

hindrance was observed for any of the serotypes. The contact residue AAV2-Q589 (in contact with AAVR-S425) is conserved in serotype 9, an aspartate in AAV1 but an alanine in most other strains.

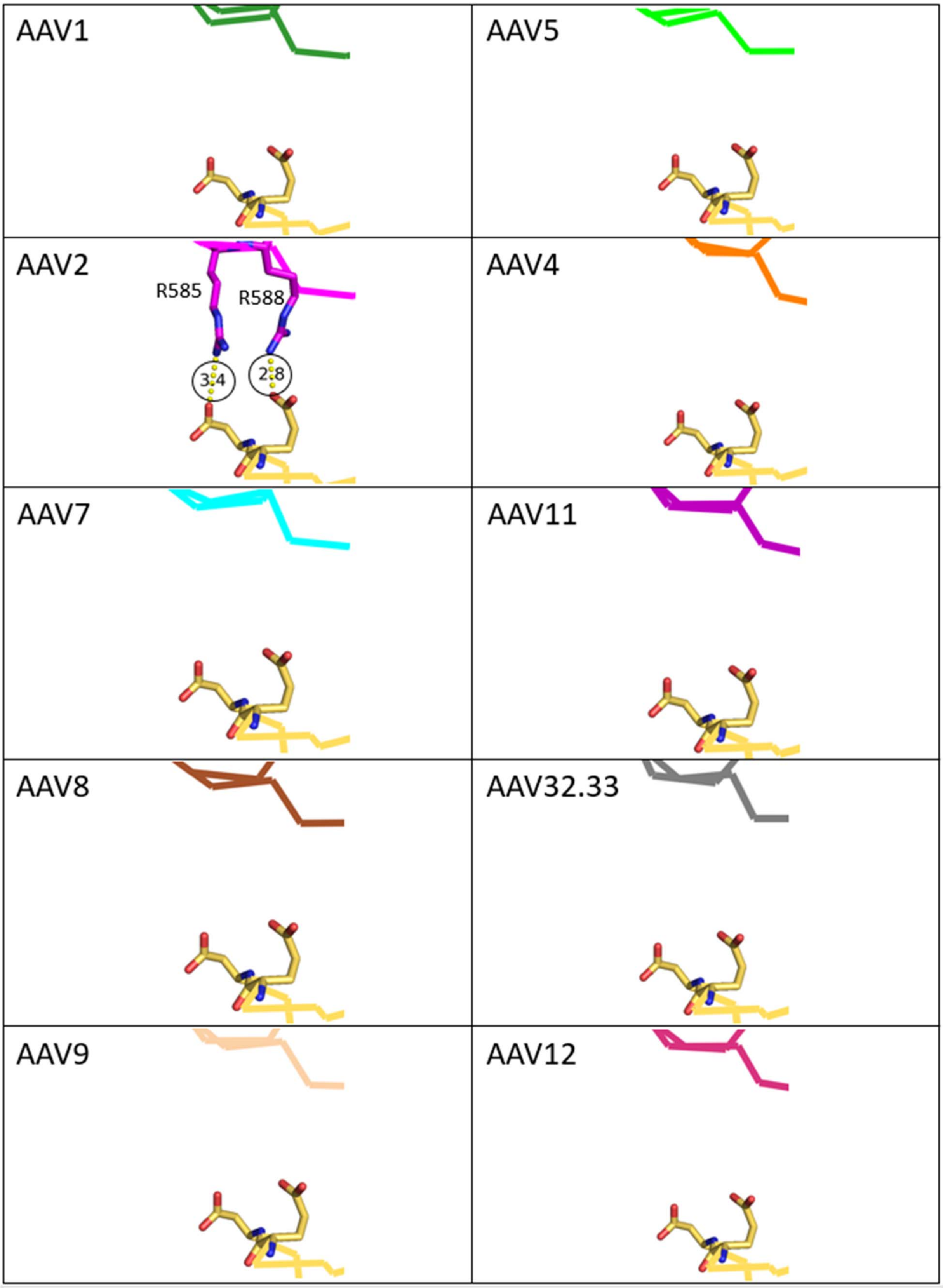
AAV1
AAV5
AAV2
R585
R588
3.4
2.8
AAV4
AAV7
AAV11
AAV8
AAV32.33
AAV9
AAV12

Figure S13 Potential interactions of variable region 8 (VR-VIII) for AAV structures after alignment to the AAV2-PKD12 receptor complex (PDBid: 6nz0). Structures and serotype color-coding are as described in Error! Reference source not found., and AAVR is shown with yellow carbons. Interactions of a symmetry-equivalent VR-VIII with a different region of PKD2 are shown in Error! Reference source not found.. Neither of the AAV2 contact residues, R585 nor R588 (interacting with AAVR D459 and E458 respectively), are conserved outside clade B. None of the serotypes analysed would conflict with PKD2 as bound in AAV2.

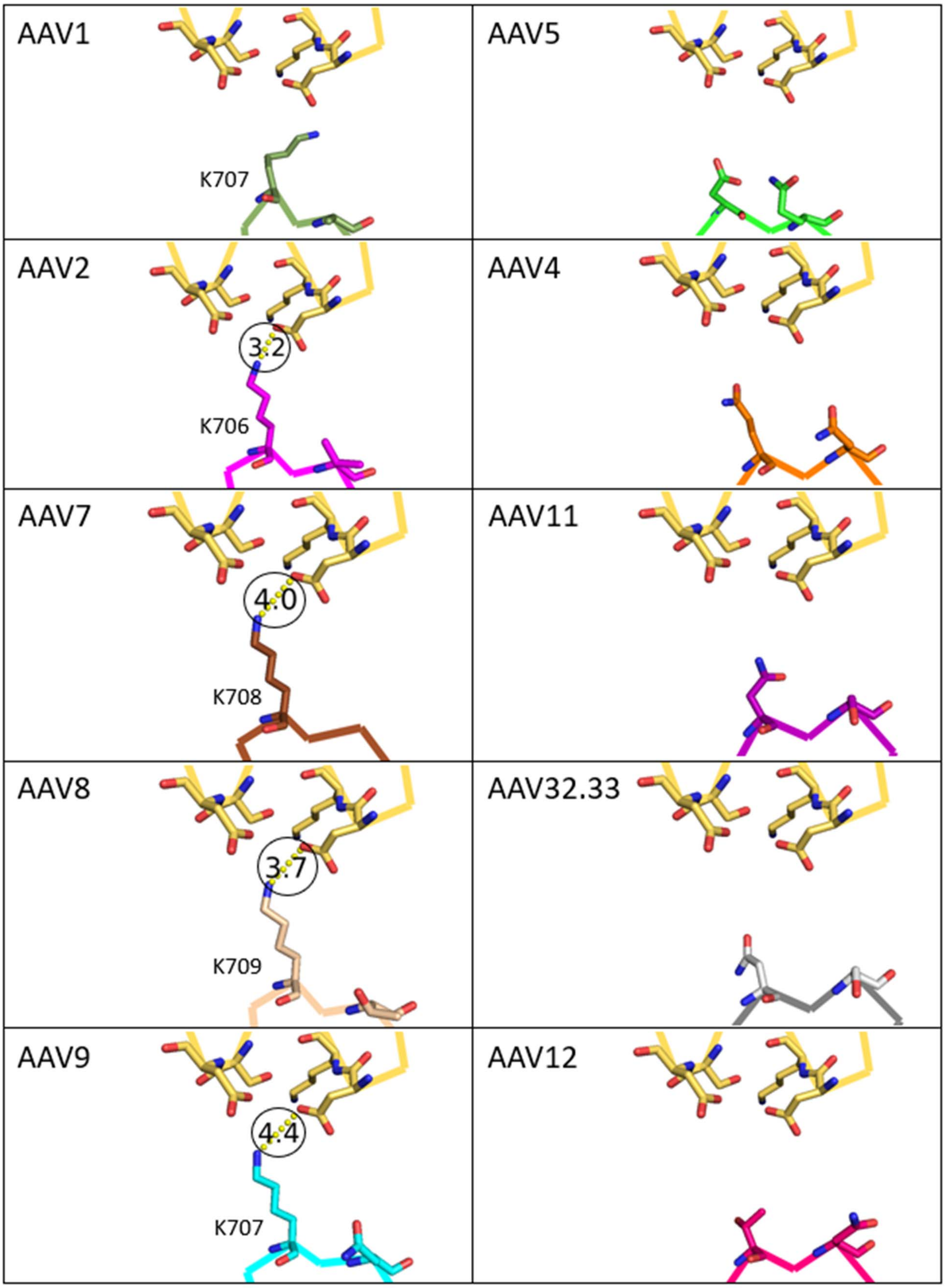
AAV1
K707
AAV5
AAV2
3.2
K706
AAV4
AAV7
4.0
K708
AAV11
AAV8
3.7
K709
AAV32.33
AAV9
4.4
K707
AAV12

Figure S14 Potential interactions of variable region 9 (VR-IX) for AAV structures after alignment to the AAV2-PKD12 receptor complex (PDBid: 6nz0). Structures and serotype color-coding are as described in Error! Reference source not found., and AAVR is shown with yellow carbons. There would be no steric hindrance for this region between PKD2 and these serotypes. However, the positive charge located at AAV2-K706 (and which is conserved in serotypes AAV1, 7, 8, 9) is not conserved within the AAV5 and AAV4-clade members.

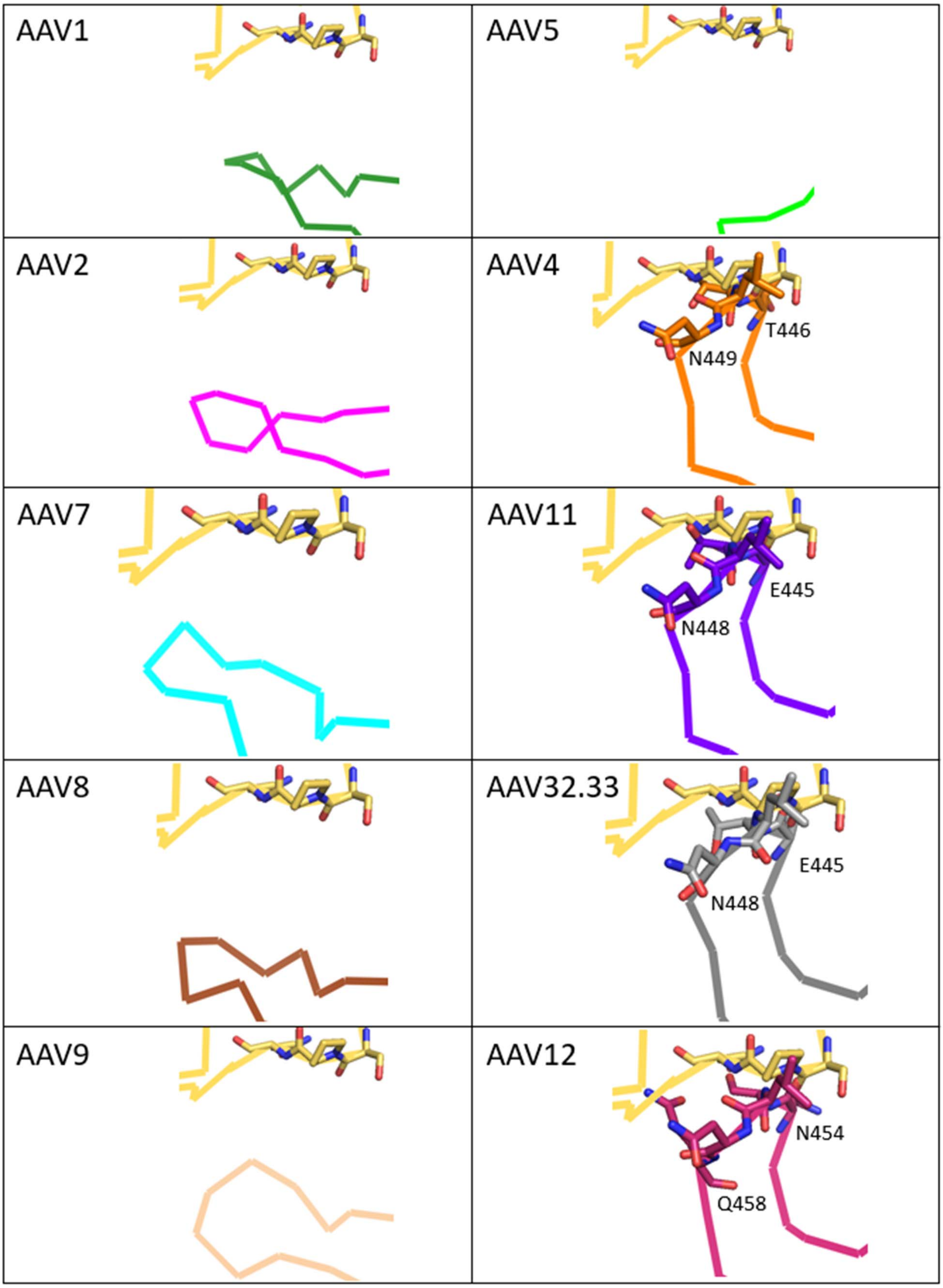
AAV1
AAV5
AAV2
AAV4
T446
N449
AAV7
AAV11
E445
N448
AAV8
AAV32.33
E445
N448
AAV9
AAV12
N454
Q458

Figure S15 Potential interactions of variable region 4 (VR-IV) for AAV structures after alignment to the AAV2-PKD12 receptor complex (PDBid: 6nz0). Structures and serotype color-coding are as described in Error! Reference source not found., and AAVR is shown with yellow carbons. A strong steric hindrance was observed between PKD2 (S413, P414 and Q415) and VR-IV for all the AAV4-clade members (specifically for AAV4, T446 through N449).

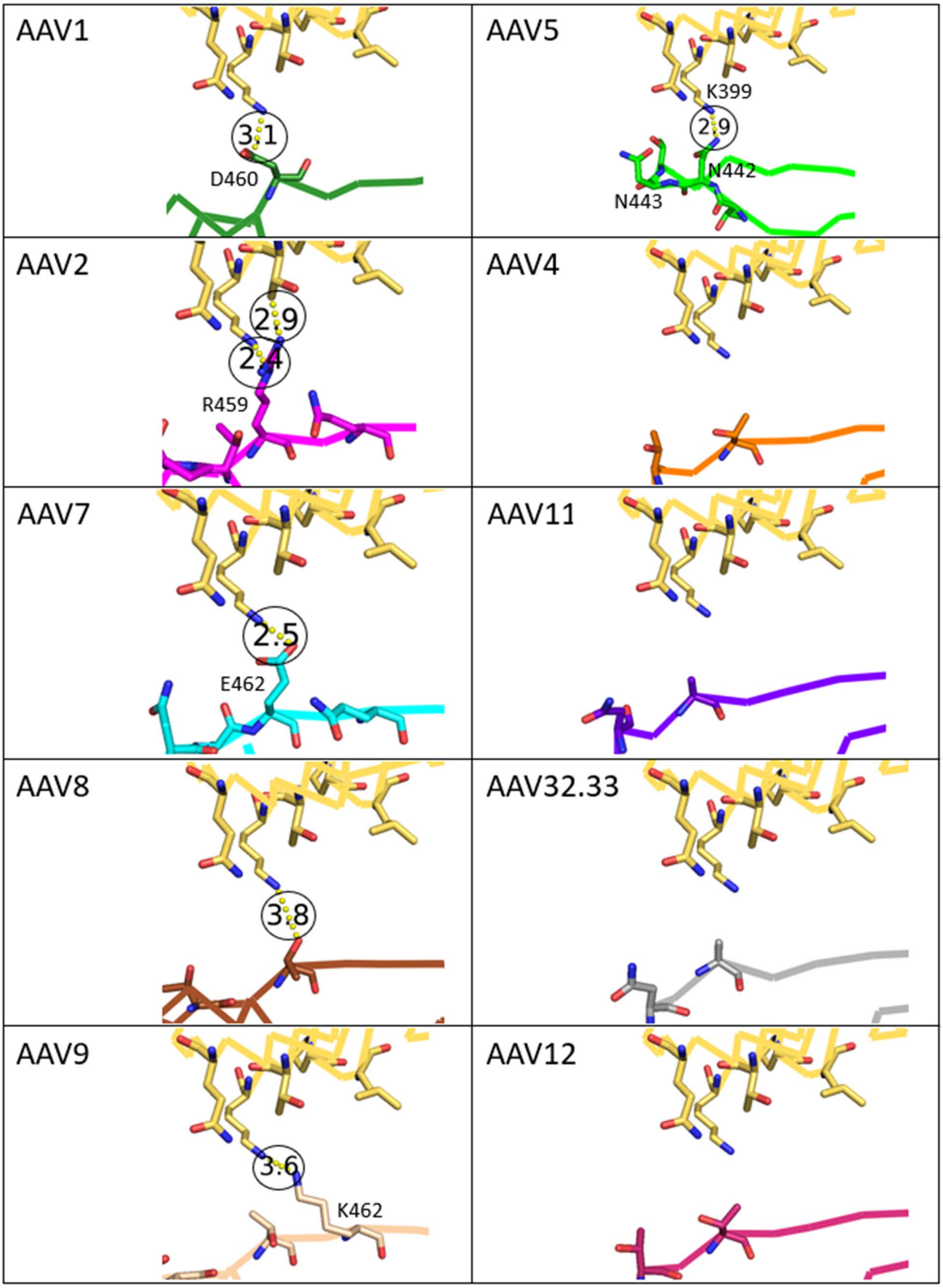
AAV1
3.1
D460
AAV5
K399
2.9
N442
N443
AAV2
2.9
2.4
R459
AAV4
AAV7
2.5
E462
AAV11
AAV8
3.8
AAV32.33
AAV9
3.6
K462
AAV12

Figure S16 Potential interactions of variable region 4 (VR-IV) for AAV structures after alignment to the AAV5-PKD12 receptor complex (PDBid: 7kpn Structures and serotype color-coding are as described in Error! Reference source not found., and AAVR is shown with yellow carbons. A steric hindrance, in addition to an electrostatic repulsion, is likely between AAVR-K399 (yellow) and AAV2-R459 (magenta) or AAV9-K462 (wheat).

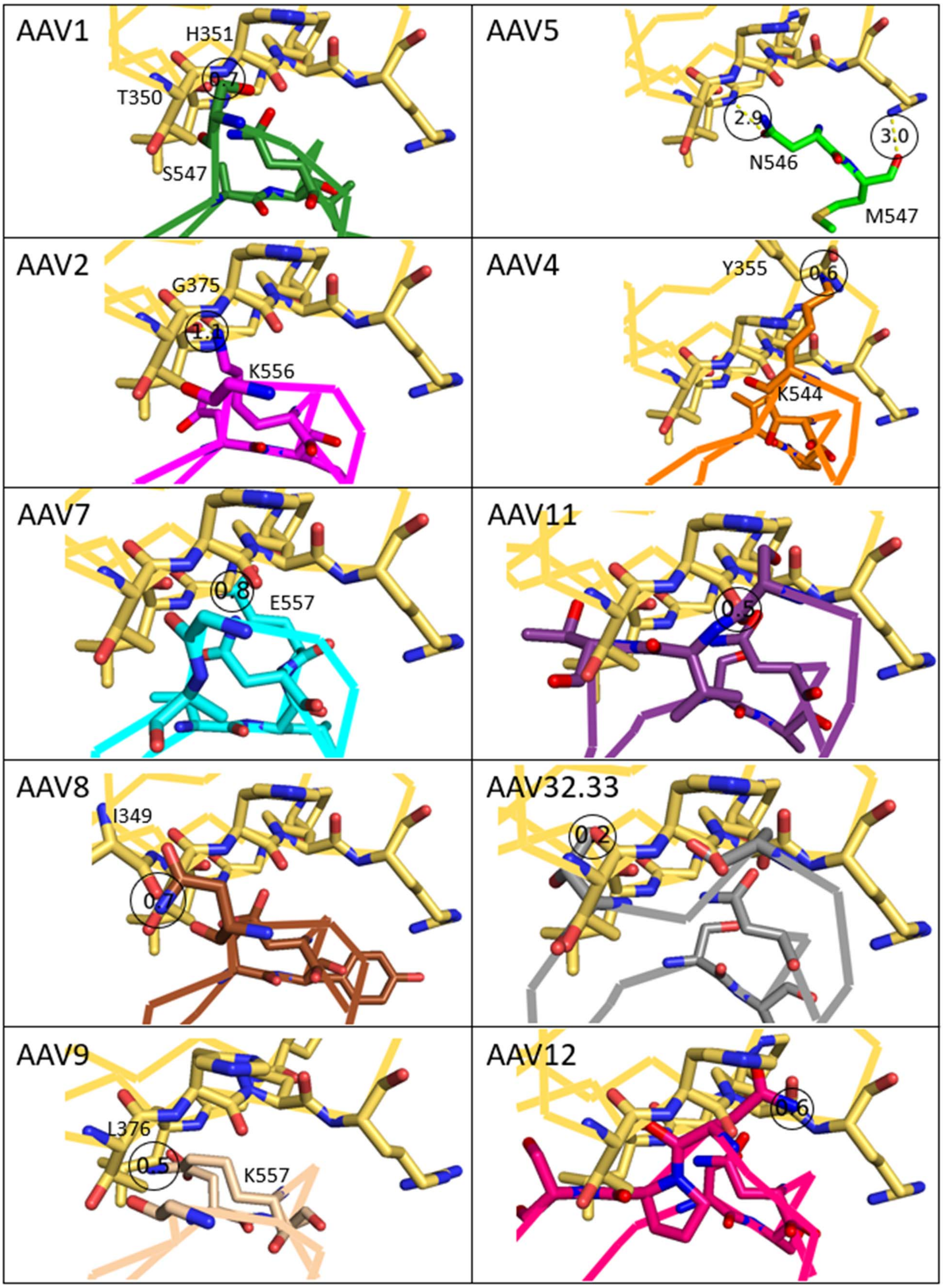
AAV1
H351
0.7
T350
S547
AAV5
2.9
3.0
N546
M547
AAV2
G375
1.1
K556
AAV4
Y355
0.6
K544
AAV7
0.8
E557
AAV11
0.5
AAV8
I349
0.7
AAV32.33
0.2
AAV9
I376
0.5
K557
AAV12
0.6

Figure S17 Potential interactions of variable region 7 (VR-VII) for AAV structures after alignment to the AAV5-PKD12 receptor complex (PDBid: 7kpn). Structures and serotype color-coding are as described in Error! Reference source not found., and AAVR is shown with yellow carbons. A steric clash is observed between AAV4-K544 and AAVR-Y355 and multiple conflicts for the other AAV4-clade members. For serotypes other than AAV5, this region contains multiple contacts that would be incompatible to forming a complex with PKD1.

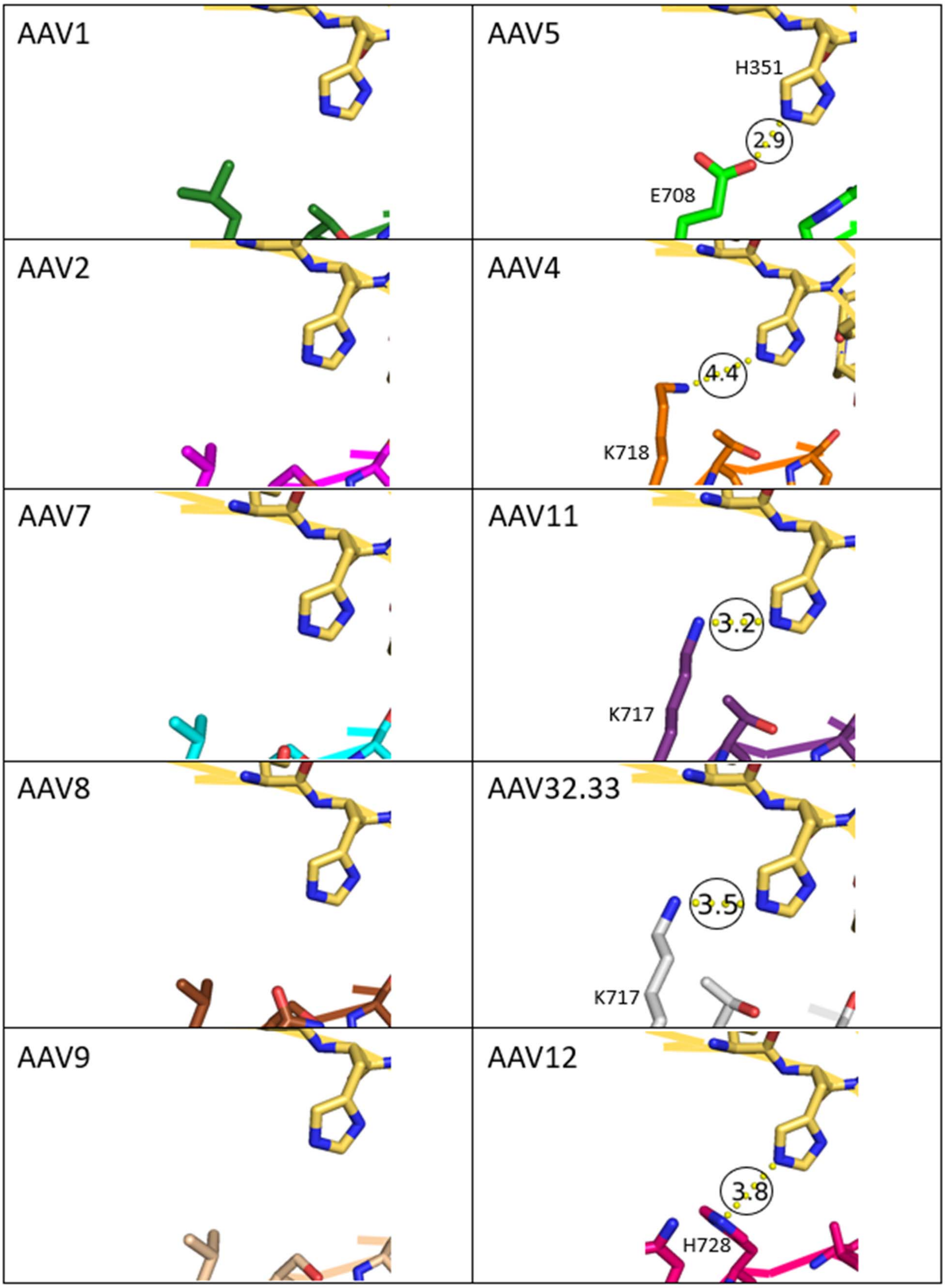
AAV1
AAV5
H351
2.9
E708
AAV2
AAV4
4.4
K718
AAV7
AAV11
3.2
K717
AAV8
AAV32.33
3.5
K717
AAV9
AAV12
3.8
H728

Figure S18 Potential interactions of variable region 9 (VR-IX) for AAV structures after alignment to the AAV5-PKD12 receptor complex (PDBid: 7kpn). Structures and serotype color-coding are as described in Error! Reference source not found., and AAVR is shown with yellow carbons. No steric hindrance was observed for this region between PKD1 and these serotypes. However, there would likely be unfavorable electrostatics for the AAV4-clade (between AAVR-H351 and AAV4-K718 or AAV12-H728) replacing the salt bridge in AAV5 (E708).

	Variable regions								
	I	II	III	IV	V	VI	VII	VIII	IX
AAV1	C		C		C	C		C	C
AAV6.1									
AAV2	C		C		C	C		C	C
AAV3b									
AAV7	X							Non-conserved	
AAV8	X							Partially conserved	
AAV9	X					Partially conserved		Conserved	
AAV4	X		X	X	X				Non-conserved
AAV11	X		X	X	X				Non-conserved
AAVrh32.33	X		X	X	X				Non-conserved
AAV12	X		X	X	X				Non-conserved
AAV5	X		X						R

Binds to PKD2; Does not bind PKD2; C: Contact residue; X: steric clash; R: electrostatic repulsion

Figure S19 Summary of interactions between PKD2 and various serotypes.

	Variable regions								
	**I**	**II**	**III**	**IV**	**V**	**VI**	**VII**	**VIII**	**IX**
AAV1							X		
AAV6.1									
AAV2				X/R			X		
AAV3b									
AAV7							X		
AAV8							X		
AAV9				X/R			X		
AAV4							X		R
AAV11							X		R
AAVrh32.33							X		R
AAV12							X		R
AAV5				C			C		C

Binds to PKD1; Does not bind PKD1; C: Contact residue; X: steric clash; R: electrostatic repulsion

Figure S20 Summary of interactions between PKD1 and various serotypes.
